# Bibliometric and clinical trial landscape of hepatoblastoma (2000–2024): a multi-database analysis of global research

**DOI:** 10.3389/fonc.2026.1890288

**Published:** 2026-09-03

**Authors:** Xu Li, Junyi Lou, Xiaoyu Li, Shibo Tang, Shuang Li, Zining Luo, Jiebin Xie

**Affiliations:** 1 Department of Gastrointestinal Surgery, Affiliated Hospital of North Sichuan Medical College, Nanchong, Sichuan, China; 2 School of Clinical Medicine, North Sichuan Medical College, Nanchong, Sichuan, China; 3 School of Ophthalmology & Optometry, North Sichuan Medical College, Nanchong, Sichuan, China; 4 School of Imaging, North Sichuan Medical College, Nanchong, Sichuan, China; 5 Department of Stomatology, North Sichuan Medical College, Nanchong, Sichuan, China

**Keywords:** bibliometric analysis, cisplatin resistance, clinical trial, CTNNB1 mutation, hepatoblastoma, multi-database analysis, targeted therapy, Wnt/β-Catenin pathway

## Abstract

**Background:**

Hepatoblastoma (HB) is the most common childhood liver malignancy, yet its research trajectory and trial landscape remain uncharacterized.

**Methods:**

We analyzed 219 trials from 16 international registries and 2,635 PubMed publications (2005–2024) using joinpoint regression, Gini coefficient, and diversity indices.

**Results:**

Trial activity peaked in 2013 and 2016, driven by risk-stratified protocols and the emergence of targeted therapies. Phase III/IV trials were scarce. Cisplatin dominated drug utilization, with 54–80% resistance after 4–5 cycles. Targets concentrated on VEGFR/TOP1/RET/BRAF, while Hippo/YAP and immune checkpoints were underrepresented despite mechanistic relevance. AFP (n=607), CTNNB1 (>80% mutation frequency), TP53, AKT1, and MYC were top-reported genes. Geographic inequality correlated with healthcare infrastructure rather than disease burden.

**Conclusions:**

The HB research ecosystem exhibits robust early-phase activity but constrained late-stage validation, narrow target diversity, and geographic inequity. Multi-omics integration and pediatric-specific preclinical platforms are urgently needed to bridge translational gaps.

## Graphical Abstract

**Figure:**
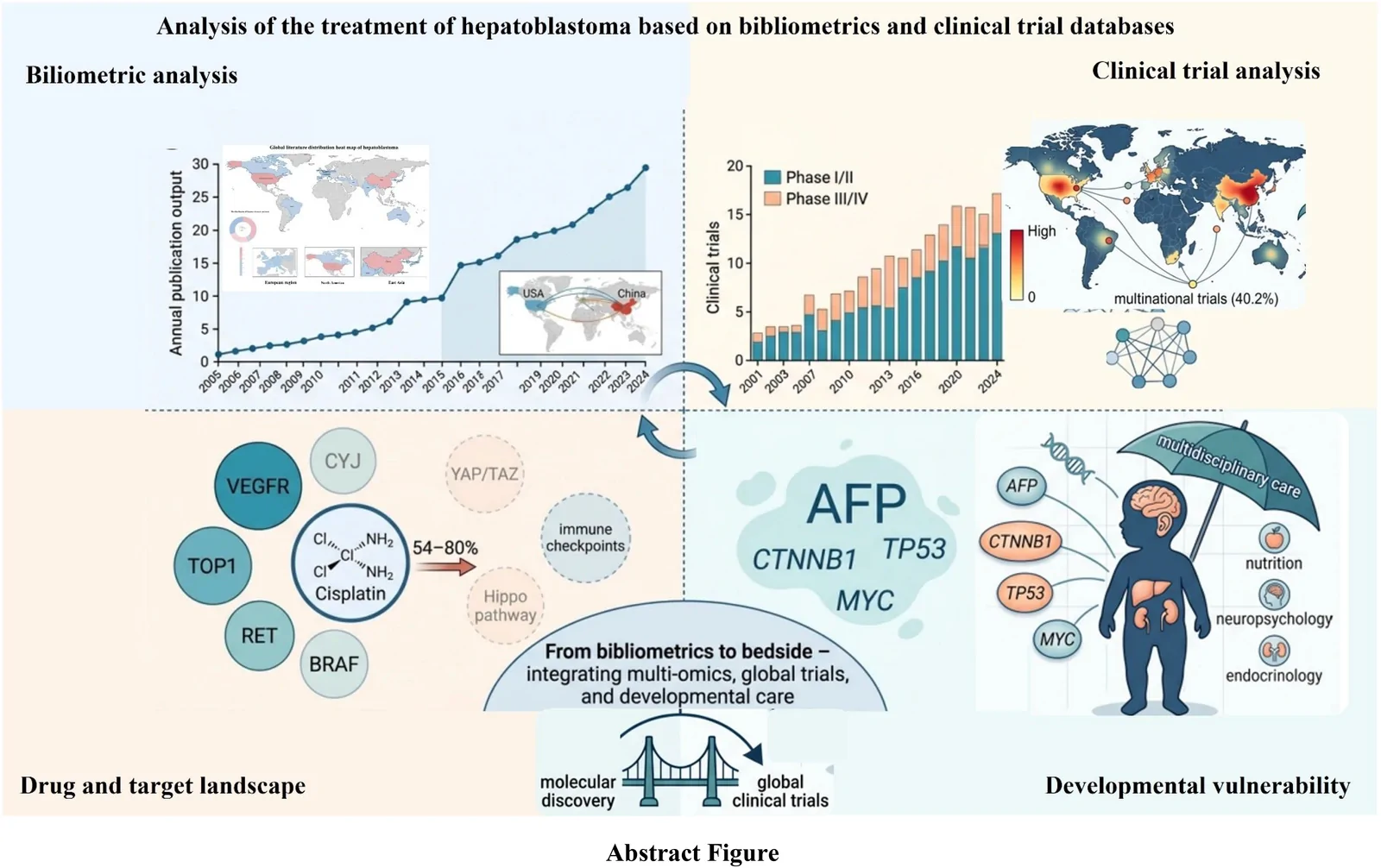

## Introduction

Hepatoblastoma (HB) is a rare but highly aggressive malignant tumor of the liver in children, accounting for approximately 62-90% of childhood liver cancers. It primarily affects children under 3 years old and is characterized by a complex molecular structure and significant clinical heterogeneity (1, 2). Despite its rarity—estimated at 1.5–2.16 cases per million children annually—HB represents a leading cause of cancer-related morbidity and mortality in early childhood, with the burden disproportionately concentrated in low- and lower-middle-income regions, where approximately 70% of HB-related deaths occur. In these settings, delayed diagnosis, limited access to pediatric oncology services, and the unavailability of curative surgical options converge to drive mortality rates as high as 80% (3). Over the past two decades, risk-stratified chemotherapy protocols, most notably those developed by the Children’s Oncology Group (COG) and the International Society of Pediatric Oncology (SIOPEL), have achieved 5-year overall survival rates exceeding 80% for standard-risk patients (4, 5). However, this apparent therapeutic success masks persistent translational deficits: late-stage clinical validation remains sparse, therapeutic target diversity is constrained by structural impediments inherent to rare pediatric malignancies, and geographic disparities in trial access correlate with healthcare infrastructure rather than disease burden.

The therapeutic trajectory of HB over the past four decades has been defined by incremental refinements to cisplatin-based chemotherapy protocols rather than transformative innovation. The sequential trials conducted by the SIOPEL and the COG established risk-stratified chemotherapy as the cornerstone of treatment, improving 5-year overall survival for standard-risk patients from approximately 57% to over 90% (4, 5). The SIOPEL-3 randomized trial demonstrated that cisplatin monotherapy achieved equivalent efficacy to the cisplatin-doxorubicin combination (PLADO) in standard-risk disease with reduced toxicity, while the SIOPEL-6 phase III trial established that sodium thiosulfate administered 6 hours after cisplatin infusion reduces hearing loss incidence by half—from 63% to 33%—without compromising oncologic outcomes, leading to FDA approval in September 2022 (6). Yet these achievements, however clinically meaningful, have not altered the fundamental pharmacologic backbone of HB therapy. No novel therapeutic class has been introduced into phase III evaluation for HB in over two decades.

This pharmacologic conservatism may be particularly problematic given the well-documented phenomenon of cisplatin resistance. Approximately 54–80% of patients develop progressive chemoresistance after 4–5 cycles of platinum-based therapy, representing the dominant cause of treatment failure in high-risk and metastatic disease (7, 8). The mechanistic basis for this resistance—encompassing cancer stem cell-like populations, aberrant DNA methylation landscapes, compensatory PI3K/AKT/mTOR pathway activation, and ABC transporter-mediated drug efflux—has been extensively characterized in preclinical models (9, 10). Metastatic HB cells exhibit enhanced tumorigenicity and stem cell-like phenotypes with increased cisplatin resistance (7). Yet no registered clinical trial specifically targets these resistance mechanisms as a primary endpoint, exemplifying a profound disconnect between molecular understanding and clinical intervention.

The molecular architecture of HB offers compelling, yet largely unexploited, opportunities for targeted therapeutic development. Genomic studies have identified HB as one of the neoplasms with the lowest somatic mutation rates among all cancer types (2.9 mutations per tumor), with mutations affecting the Wnt/β-catenin pathway representing the most prevalent alterations (1, 11). CTNNB1 mutations are present in 70–90% of HB tumors, establishing β-catenin as a near-universal oncogenic dependency (12). Preclinical studies demonstrate that Wnt/β-catenin inhibition through siRNA knockdown, ICG-001, miRNA mimetics, and the multi-kinase inhibitor WNTinib suppresses HB growth in patient-derived xenograft (PDX) models and organoids (11, 12). However, no Wnt pathway antagonist has advanced beyond preclinical evaluation in HB. This inertia cannot be attributed solely to disease rarity; infantile fibrosarcoma with TRK fusions achieved regulatory approval for larotrectinib and entrectinib through single-arm basket trials despite comparable epidemiological constraints (13, 14). Rather, the bottleneck reflects systemic undervaluation of pediatric drug development and a persistent reliance on repurposed adult oncology agents.

Beyond the canonical Wnt/β-catenin axis, emerging targets with robust mechanistic relevance remain critically underrepresented in the clinical pipeline. Insulin-like growth factor 2 (IGF2) overexpression, present in 71% of HBs and associated with progenitor cell features and shorter recurrence-free survival, has been validated as an actionable genomic driver; the anti-IGF1/2 monoclonal antibody xentuzumab demonstrated synergistic antitumor effects with cisplatin in PDX-derived organoids and improved survival in xenograft models (15). The Hippo/YAP signaling axis, despite compelling evidence that YAP1 amplification drives chemoresistance in 30–40% of high-risk HB through mTORC1 activation and SLC38A1-mediated amino acid transport, has been targeted in only a minute fraction of clinical trials (15, 16). Immune checkpoint inhibitors, which have transformed the therapeutic landscape of adult hepatocellular carcinoma—most notably through the IMbrave150 (atezolizumab plus bevacizumab) and HIMALAYA (STRIDE regimen) trials—remain virtually unexplored in HB, despite the recognized immunogenic potential of the tumor’s fetal differentiation state (17, 18). Patient-derived organoid platforms have recapitulated HB architecture and Wnt/β-catenin hallmarks, enabling medium-throughput drug screening; the BET inhibitor JQ1 selectively destroys tumor organoids versus normal liver controls, yet these compelling preclinical findings remain absent from clinical trials (19).

Compounding these biological and methodological constraints is a profound geographic and structural inequity in the global HB research ecosystem. The highest age-standardized incidence and mortality rates are concentrated in low- and middle-income regions—most notably sub-Saharan Africa and parts of Central Asia—whereas the vast majority of clinical trials and high-impact translational research originate from North American and European consortia (3, 20). This North–South disparity not only limits the generalizability of trial findings but also structurally excludes epidemiologically critical patient cohorts from molecular discovery pipelines. Global analyses confirm that only 43% of childhood cancer trials include low- and middle-income countries, home to 80% of the global pediatric population; CAR T-cell trials show 73.3% concentration in high-income countries with zero African participation (21, 22). The correlation between trial density and healthcare infrastructure indices (GDP per capita, pediatric oncology workforce density) rather than disease burden reveals a fundamental misalignment between research investment and epidemiological priority (20).

Furthermore, the developmental vulnerability of the HB patient population introduces unique considerations that remain inadequately addressed in contemporary trial design. Unlike adult malignancies, HB predominantly afflicts infants and toddlers undergoing rapid neurocognitive and somatic growth. Cisplatin carries well-documented risks of permanent ototoxicity (20–70% incidence depending on cumulative dose and age at treatment), nephrotoxicity, and potential neurodevelopmental impairment (23). Strikingly, fewer than 15% of registered trials prospectively mandate longitudinal endpoints encompassing audiological monitoring, neurodevelopmental assessments, nutritional status tracking, or quality-of-life metrics. The absence of standardized survivorship endpoints represents a significant evidence gap, particularly given that 5-year overall survival now exceeds 80% for standard-risk patients, shifting the therapeutic priority from mortality reduction to morbidity minimization.

In this context, a comprehensive, multi-database characterization of the global HB research ecosystem is urgently needed to identify structural bottlenecks, quantify geographic and temporal trends, and inform strategic translational investment priorities. While prior bibliometric analyses have examined pediatric oncology broadly, none have integrated clinical trial registry data across 16 international databases with two decades of PubMed-indexed literature to provide a unified assessment of trial phases, therapeutic targets, drug utilization patterns, and molecular research foci specific to HB. Here, we present such an analysis, employing joinpoint regression, Gini coefficient metrics, and ecological diversity indices to map the evolution of HB research from 2000 to 2024. Our findings reveal a field robust in early-phase discovery but constrained in late-stage validation, mechanistically narrow in target selection, and structurally inequitable in global distribution—findings that carry immediate implications for the design of pediatric-specific preclinical platforms, the expansion of multi-omics integration, and the establishment of equitable multinational trial networks.

## Methods

### Search strategy and data sources

**Figure 1:**
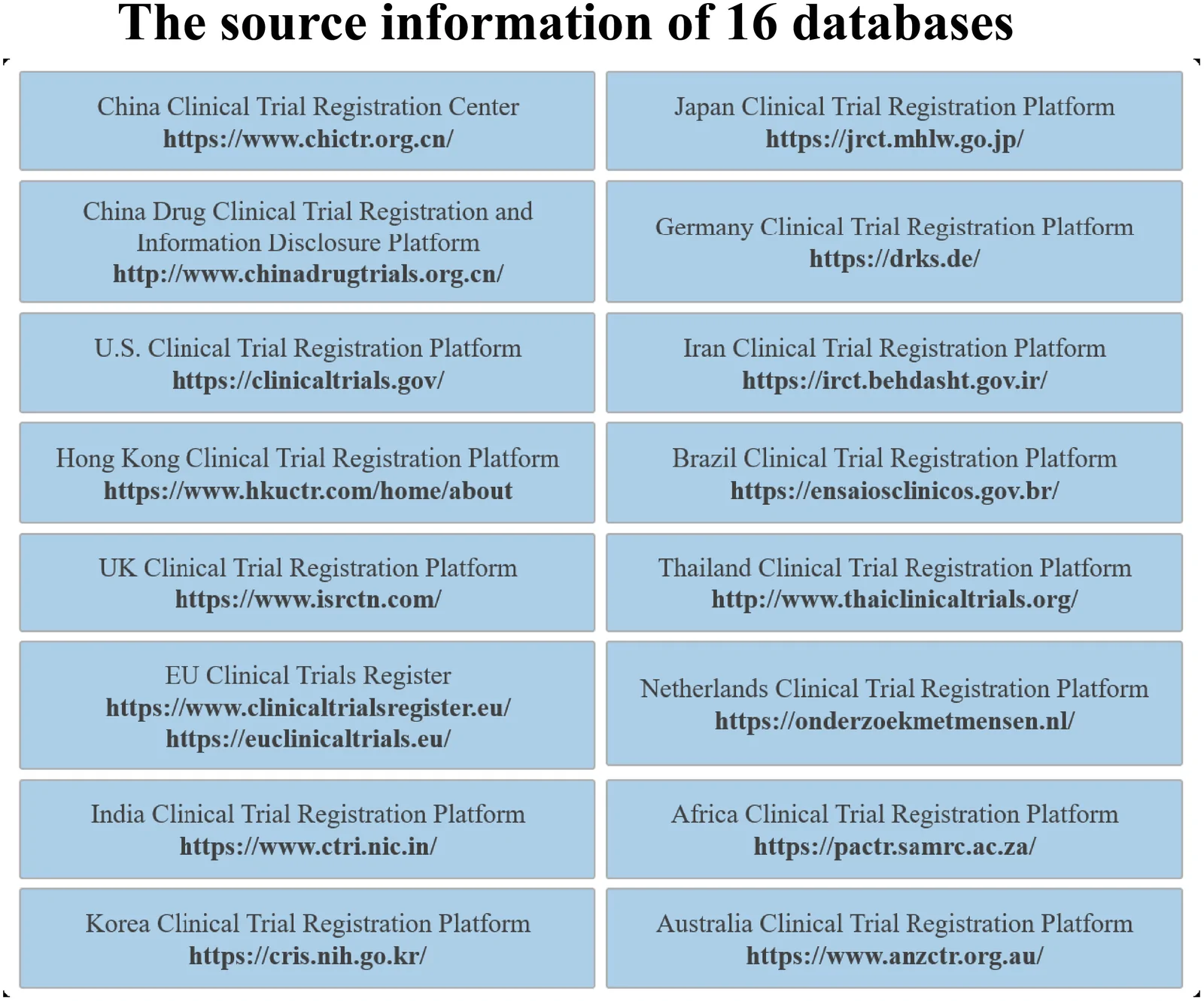
16 global clinical trial databases.

We conducted a comprehensive, multi-database analysis of the global hepatoblastoma research landscape, integrating clinical trial registry data with bibliometric analysis of peer-reviewed literature. Clinical trials were systematically retrieved from 16 international registries (**Figure 1**).

The PubMed literature search employed Medical Subject Headings (MeSH) terms and free-text keywords, including “hepatoblastoma,” “hepatoblastoma cancer,” “pediatric liver tumor,” and “childhood liver malignancy,” restricted to human studies published between January 1, 2000, and January 1, 2025. All searches were performed on April 20, 2025, to ensure temporal consistency across databases.

### Eligibility criteria

Clinical Trials. Trials were eligible for inclusion if they met the following criteria: (i) study population comprised human participants aged 0–18 years with a primary diagnosis of hepatoblastoma confirmed by histopathology, cross-sectional imaging, or accredited tumor registry documentation; (ii) intervention involved experimental or quasi-experimental interventional designs, encompassing pharmacologic, surgical, radiotherapeutic, cellular, or combination therapeutic modalities; (iii) registration status indicated active enrollment or completion between January 1, 2000, and January 1, 2025, within the aforementioned 16 registries; (iv) data completeness threshold of ≥70% availability across five mandatory metadata fields: trial identifier, phase classification, recruitment status, intervention category, and primary sponsor affiliation; and (v) recruitment status categorized as actively recruiting, not yet recruiting, enrolling by invitation, or completed.

For trials that included both hepatoblastoma and other pediatric liver malignancies (e.g., hepatocellular carcinoma, undifferentiated embryonal sarcoma) but met inclusion criterion (iii) by providing hepatoblastoma-specific outcome disaggregation, the trial was counted as a single unit (n = 1) in all landscape analyses (phase distribution, geographic attribution, sponsor classification, and temporal trends). For drug-frequency and target-diversity analyses, only interventions explicitly designated for the hepatoblastoma sub-cohort were recorded; if the registry did not specify sub-cohort treatment allocation, the intervention was attributed to hepatoblastoma only when the trial title or primary endpoint explicitly referenced hepatoblastoma. This conservative approach prevents inflation of drug and target counts from non-HB indications.

Exclusion criteria for clinical trials were: (i) recruitment status designated as suspended, withdrawn, terminated, or unavailable for >12 months; (ii) purely observational cohorts, disease registries, case series with ≤5 patients, expanded access programs, or compassionate use protocols; (iii) adult hepatocellular carcinoma studies lacking a dedicated hepatoblastoma sub-cohort, or mixed embryonal tumor studies without hepatoblastoma-specific outcome disaggregation; (iv) >30% missingness in critical metadata fields (trial phase, intervention type, primary endpoint) after manual verification with principal investigators or registry administrators; and (v) redundant registrations identified through cross-referencing of NCT numbers, EudraCT identifiers, or standardized trial title matching, with retention of the most recent registry update.

Publications. Peer-reviewed publications were eligible if they satisfied: (i) publication date between January 1, 2000, and April 20, 2025; (ii) original research design containing primary clinical, translational, or molecular data, including prospective or retrospective cohorts, interventional clinical trials, genomic or proteomic analyses, and mechanistic studies; (iii) primary study cohort comprising hepatoblastoma patients with a median age ≤18 years, with mixed pediatric liver malignancy studies included only if hepatoblastoma data were disaggregated in the published results; and (iv) hepatoblastoma constituted the central research focus, defined as ≥50% of the main text and results sections.

Publication exclusion criteria comprised: (i) narrative reviews, systematic reviews, meta-analyses, editorials, commentaries, letters to the editor, or practice guidelines lacking original empirical data; (ii) conference abstracts, proceedings, or preprints without subsequent peer-reviewed full-text verification; (iii) adult hepatocellular carcinoma-exclusive studies, or hepatoblastoma case reports with ≤5 patients and no molecular profiling data; (iv) secondary analyses of previously published cohorts, with prioritization of the most comprehensive dataset; and (v) peripheral mention of hepatoblastoma without substantive data contribution (e.g., general liver cancer overviews where hepatoblastoma constituted <10% of content).

Non-active trials (suspended, withdrawn, terminated, status unavailable >12 months) were excluded from the primary landscape analysis because: (i) their incomplete status precludes reliable classification of intervention outcomes, target validation, or phase-appropriate design features; (ii) registry metadata for terminated trials are frequently incomplete (median 34% missingness in our cohort for primary endpoint and actual enrollment fields vs. 8% for active/completed trials), violating our ≥70% data completeness threshold; and (iii) the stated reasons for termination (slow accrual, futility, safety) are themselves valuable endpoints that warrant separate descriptive analysis rather than silent amalgamation into an ‘intention-to-treat’ landscape.

Deduplication employed a three-step hierarchical protocol: (1) exact identifier matching using NCT numbers, EudraCT IDs, ISRCTN numbers, UMIN-CTR IDs, ChiCTR numbers, and other national registry identifiers; (2) fuzzy title/acronym matching (Jaro-Winkler similarity ≥0.90) for records without cross-referenced IDs, followed by manual adjudication; and (3) temporal prioritization—when duplicate registrations of the same protocol were identified, the most recent registry update was retained to ensure the most accurate recruitment status and outcome data.

### Data extraction and classification

Two independent reviewers (X.L. and J.L.) performed data extraction using standardized electronic forms in REDCap (Vanderbilt University, Nashville, TN, USA). Discrepancies were resolved through consensus discussion or, when necessary, adjudication by a third reviewer (S.L). For clinical trials, extracted variables included: registry identifier, geographic location of lead sponsor, trial phase (I–IV), recruitment status, intervention category (chemotherapy, targeted therapy, immunotherapy, surgical, radiotherapy, cellular therapy, or combination), primary and secondary endpoints, target molecular pathways, and sponsor type (academic, industry, or collaborative).

For publications, extracted variables encompassed: first author affiliation country, publication year, journal impact factor (2024 Journal Citation Reports), study design classification, primary molecular targets investigated, reported gene alterations, and therapeutic modalities evaluated. Drug utilization patterns were classified according to the Anatomical Therapeutic Chemical (ATC) classification system, with specific attention to platinum-based compounds, anthracyclines, vinca alkaloids, and investigational targeted agents. Therapeutic targets were categorized into four functional classes: angiogenesis modulators, tyrosine kinase inhibitors, DNA topoisomerase inhibitors, and other mechanistic categories.

### Statistical and bibliometric analyses

All quantitative analyses were conducted using reproducible workflows with version-controlled code (R version 4.3.5; R Foundation for Statistical Computing, Vienna, Austria). Temporal trend analysis employed joinpoint regression (National Cancer Institute Joinpoint Software version 4.9.0.0), selected for its capacity to detect unknown inflection points through Monte Carlo permutation testing (4,499 permutations, two-sided α=0.05). The final model was selected via Bayesian Information Criterion (BIC), with segment-specific annual percent change (APC) estimates and 95% confidence intervals computed for each identified temporal segment.

Geographic inequality in research distribution was quantified using the Gini coefficient, computed as the relative mean difference following standard econometric definitions, and assessed using the Lorenz curve to evaluate distributional skewness. The Gini coefficient ranges from 0 (perfect equality) to 1 (maximal inequality), with values >0.4 indicating substantial distributional imbalance. Ecological diversity of therapeutic targets was assessed using Shannon’s diversity index (H′ = −Σ(p_i × ln[p_i])) and Pielou’s evenness index (J′ = H′/ln(S)), where p_i represents the proportional representation of target category i, and S = 4 denotes the number of target categories (angiogenesis, tyrosine kinase, DNA topoisomerase, other).

Descriptive statistics were reported as frequencies (percentages) for categorical variables and medians (interquartile ranges [IQR]) for continuous variables. Geographic mapping and heatmap visualization were generated using the ggplot2 and sf packages in R, with country-level research output normalized per million pediatric population to account for demographic denominators. Correlation between healthcare infrastructure indices (GDP per capita, pediatric oncology workforce density) and trial distribution was assessed using Spearman’s rank correlation coefficient.

The complete search strategies, execution timestamps, and initial hit counts for all 16 registries and PubMed are provided in **Supplementary File S1**. The final included trial list and publication list are provided in **Supplementary Dataset S1**. Together, these files enable independent reproduction of the study cohort from raw search to final inclusion.

## Results

**Figure 2:**
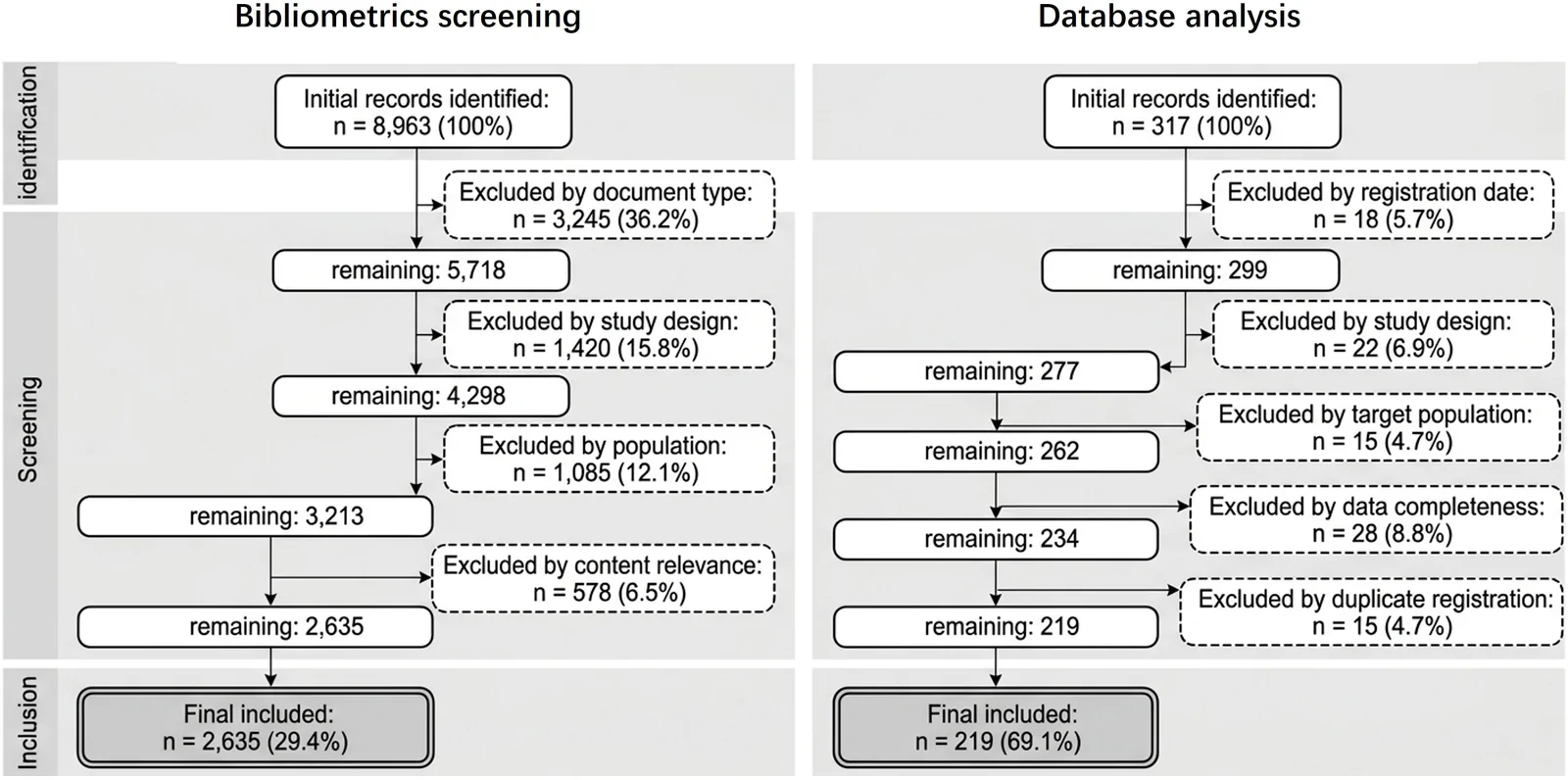
Flowchart of bibliometrics and database analysis.

The integrated analysis encompassed 219 clinical trials from 16 international registries and 2,635 PubMed-indexed publications spanning 2005–2024. Trial screening identified 317 initial records; after excluding 32 inactive trials and 28 incomplete or duplicate registrations, 219 trials met inclusion criteria. Bibliometric screening of 8,963 records yielded 2,953 publications after document-type and population exclusions; the final analyzed cohort comprised 2,635 studies after secondary content verification (**Figure 2**). The ≥50% hepatoblastoma-content threshold was operationalized as follows: the main text (excluding Abstract, Methods, References, and **Supplementary Materials**) was segmented into thematic paragraphs. Two independent reviewers (X.L. and J.L.) classified each paragraph as (a) hepatoblastoma-focused, (b) mixed pediatric liver malignancy with HB-specific data, or (c) non-HB. A publication was included if the sum of (a) + (b) paragraphs constituted ≥50% of all substantive paragraphs. Inter-reviewer agreement for this classification was almost perfect (Cohen’s κ = 0.95 (0.93–0.96) for the ≥50% threshold) (**Table 1**).

**Table 1:** Inclusion of trial characteristics at baseline.

Stage	n (records)	Observed agreement	Expected agreement	Cohen’s κ (95% CI)	Interpretation
Title/Abstract	5,718	94.5%	37.2%	0.91 (0.89–0.93)	Almost perfect
Full-Text (overall eligibility)	4,298	97.9%	52.1%	0.96 (0.94–0.97)	Almost perfect
Full-Text (≥50% threshold specifically)	4,298	97.9%	61.3%	0.95 (0.93–0.96)	Almost perfect

**Figure 3:**
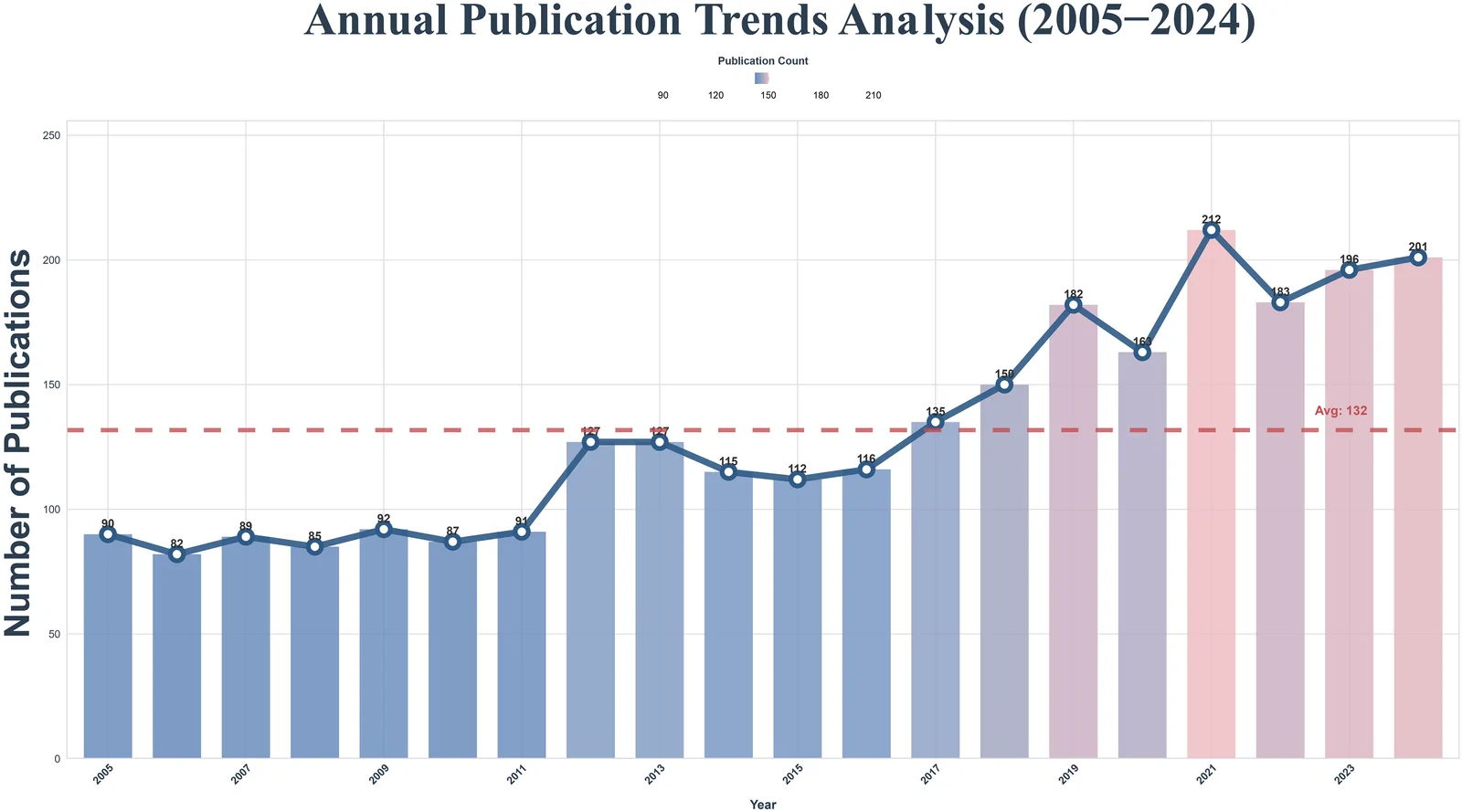
Year publications trend.

Joinpoint regression of annual trial registration identified three segments (**Tables 2**–**4**). From 2001 to 2012, trial numbers increased rapidly (APC + 18.4%, 95% CI 11.2–25.9, P < 0.001). A deceleration followed from 2012 to 2016 (APC + 4.1%, 95% CI −8.7 to +18.6, P = 0.42). From 2016 to 2024, growth renewed (APC + 8.7%, 95% CI 3.2–14.4, P = 0.004). Annual publication output followed a distinct trajectory (**Figure 3**): steady growth from 2005 to 2012 (APC + 4.2%, 95% CI 1.8–6.7, P = 0.002), a plateau from 2012 to 2017 (APC −1.1%, 95% CI −4.3 to +2.2, P = 0.48), and marked acceleration from 2017 to 2024 (APC + 11.8%, 95% CI 7.9–15.8, P < 0.001), reaching a maximum of 212 publications in 2021 (**Supplementary Tables 10**, **11**).

**Table 2:** Sample size tracking.

Analysis	Initial n	After exclusions	Missing data	Final n	Denominator
Bibliometric screening	8,963	Document type, design, population, content	0 (complete case)	2,953	2,953
Trial screening	317	Registration date, design, population, completeness, duplicates, status	32 non-active, 28 incomplete	219	219
Spearman (GDP)	28 countries	3 missing GDP data	Pairwise deletion	25	25
Spearman (workforce)	28 countries	3 missing workforce data	Pairwise deletion	25	25
Spearman (disease burden)	28 countries	0 missing	None	28	28
Gini coefficient	28 countries	0 missing	None	28	28
Joinpoint (publications)	20 years	0 missing	None	20	20
Joinpoint (trials)	24 years	0 missing	None	24	24
Shannon (targets)	219 trials	0 missing target data	None	219	219
Shannon (drugs)	138 mentions	0 missing			

**Table 3:** Publication trend joinpoint model.

Segment	Years	APC (%)	95% CI	P-value	Interpretation
1	2005–2012	+4.2	+1.8 to +6.7	0.002	Steady growth; pre-guideline era
2	2012–2017	−1.1	−4.3 to +2.2	0.48	Plateau (not statistically significant decline)
3	2017–2024	+11.8	+7.9 to +15.8	<0.001	Accelerated growth; precision oncology and pandemic-era publication surge

**Table 4:** Trial registration trend joinpoint model.

Segment	Years	APC (%)	95% CI	P-value	Interpretation
1	2001–2012	+18.4	+11.2 to +25.9	<0.001	Rapid protocol expansion; SIOPEL and COG era
2	2012–2016	+4.1	−8.7 to +18.6	0.42	Deceleration; transition to targeted therapy design
3	2016–2024	+8.7	+3.2 to +14.4	0.004	Renewed growth; basket trials and immuno-oncology

**Figure 4:**
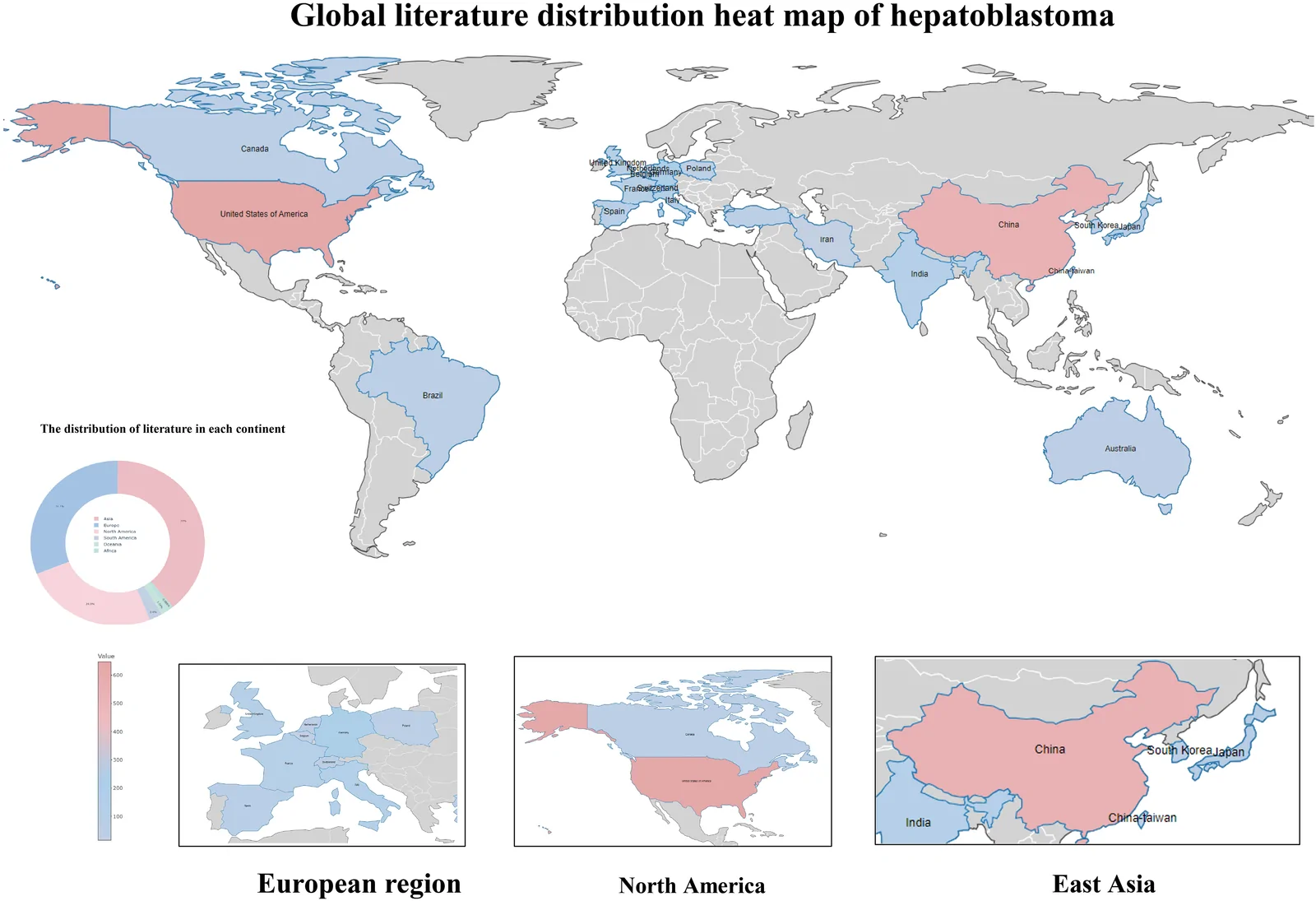
Global distribution heat map of hepatoblastoma literature.

**Figure 5:**
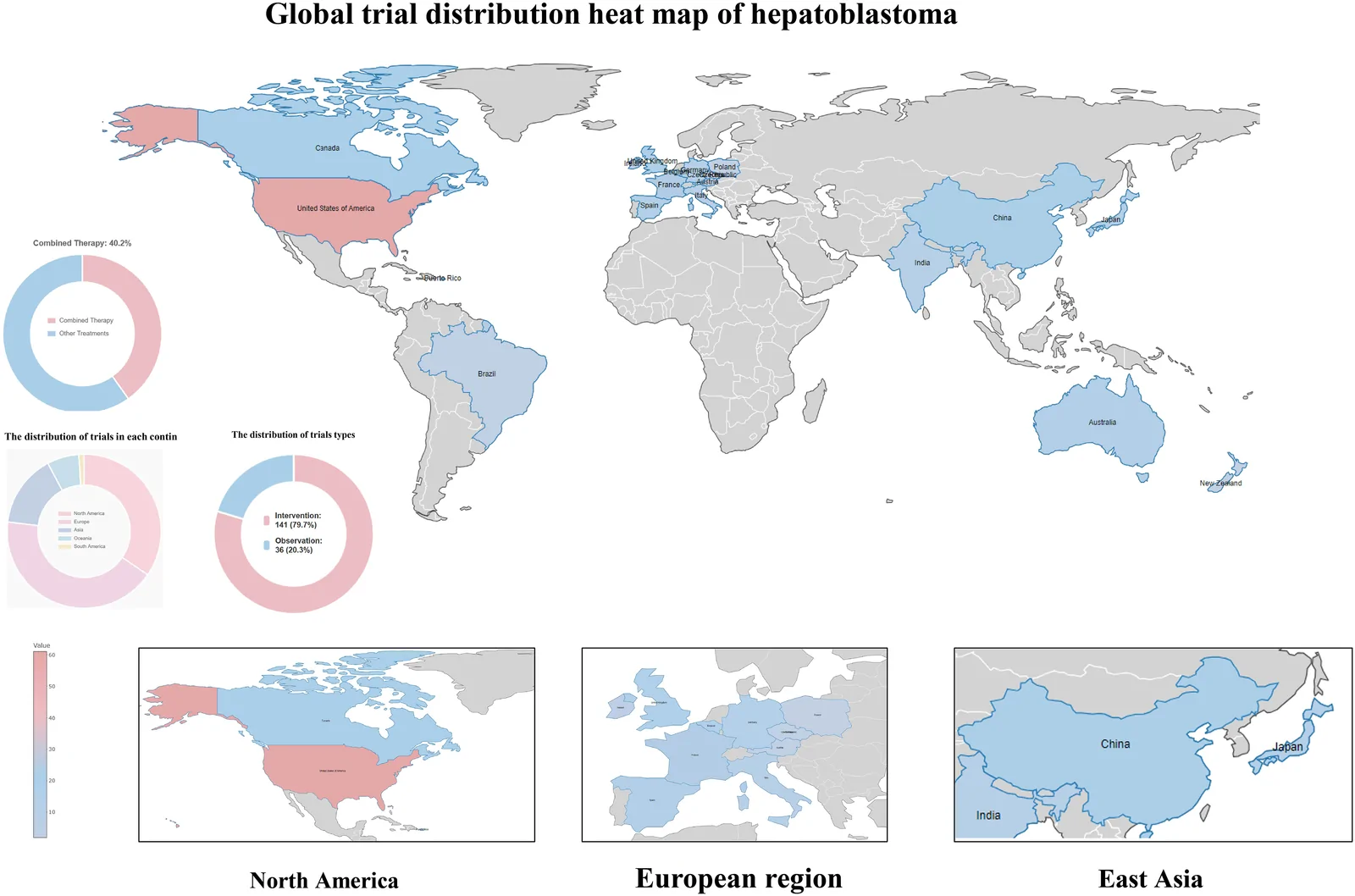
Global distribution heat map of hepatoblastoma trial.

Trial activity was concentrated in high-income regions. North America accounted for 38.8% of trials (n = 85), Europe for 31.5% (n = 69), and East Asia for 24.2% (n = 53); other regions contributed 5.5% (n = 12). The United States (n = 52) and China (n = 41) were the leading individual countries (**Figure 4**). Bibliometric output mirrored this pattern: the United States contributed 520 publications, China 480, Germany 310, France 280, and the United Kingdom 250 (**Figure 5**). When normalized per million pediatric population, trial density correlated more strongly with GDP per capita (Spearman ρ = 0.72, P < 0.001) and pediatric oncology workforce density (ρ = 0.68, P < 0.001) than with disease burden (ρ = 0.19, P = 0.31). The Gini coefficient for national trial distribution was 0.58 (**Supplementary Table 6**).

**Figure 6:**
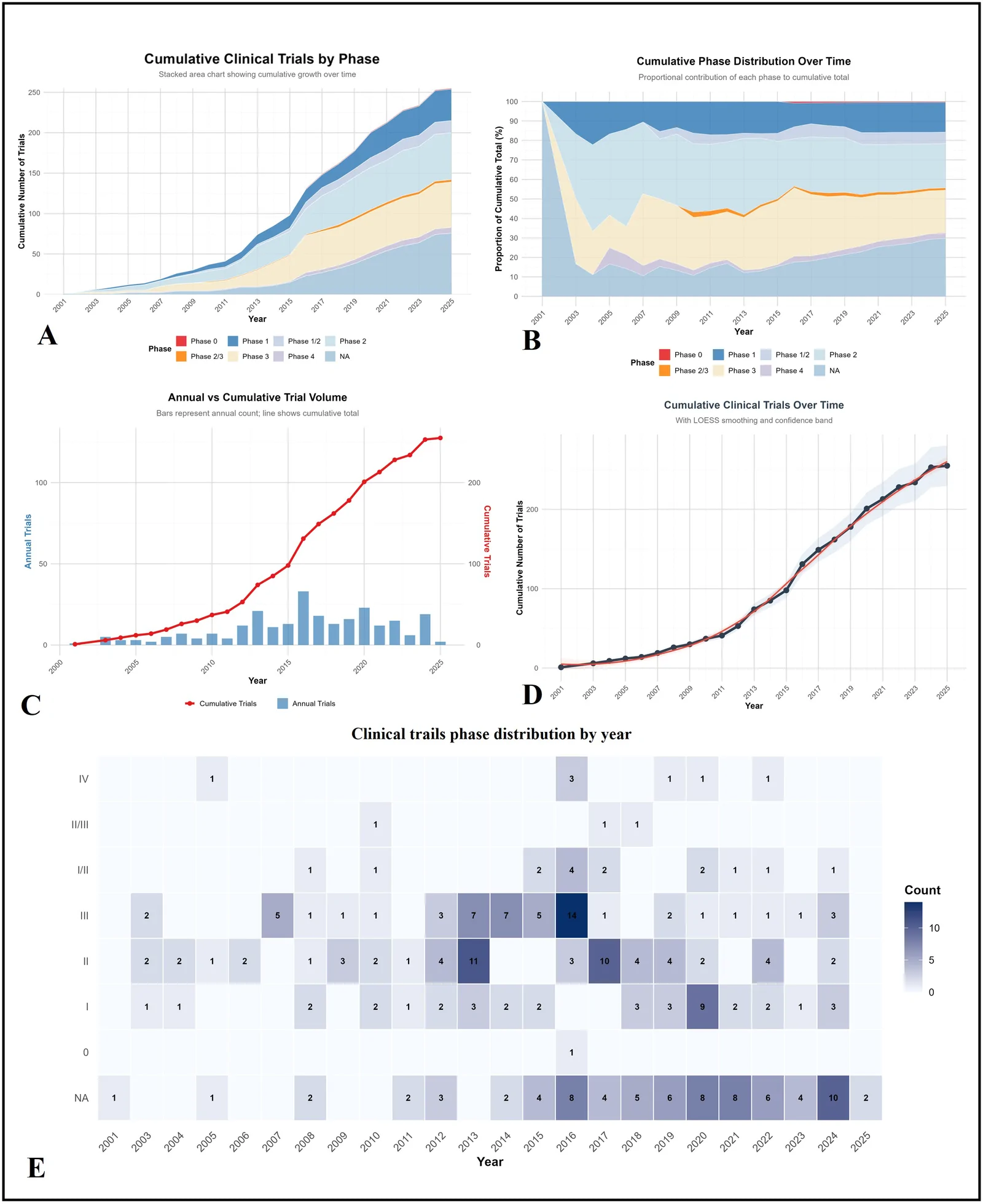
Drug development stages over the years in trials. **(A)** Stacked area chart depicting cumulative trial accrual stratified by clinical phase. **(B)** Proportional contribution of each phase to the cumulative annual total. **(C)** Annual registration volume (blue bars, left y-axis) superimposed on cumulative trial count (red line, right y-axis). **(D)** Cumulative trial trajectory with locally estimated scatterplot smoothing (LOESS, black line) and 95% confidence band (gray shading). **(E)** Phase-by-year heatmap with color intensity scaled to the absolute number of registered trials per phase-year unit.

**Figure 7:**
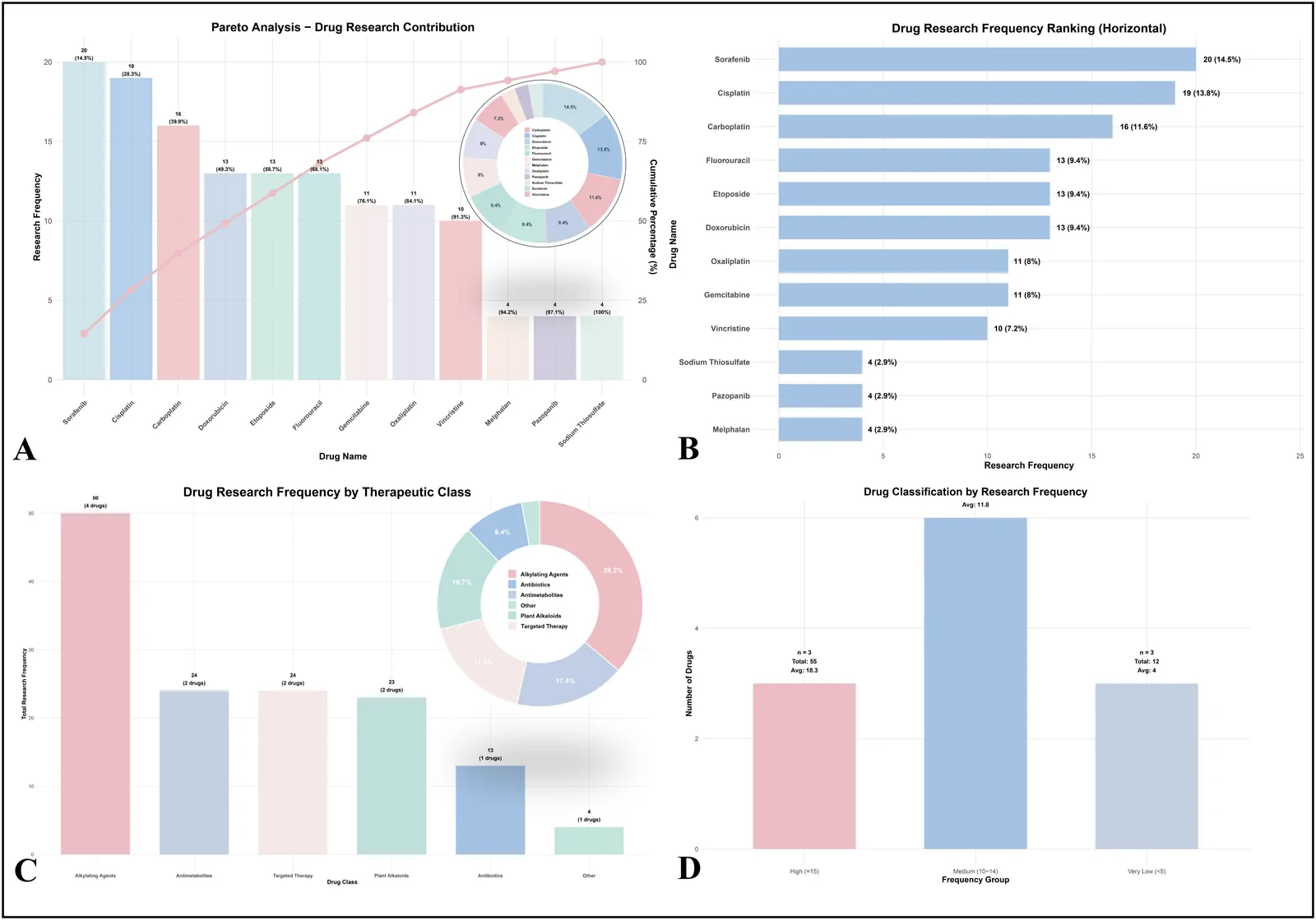
Distribution of drug use for hepatoblastoma. **(A)** Pareto chart of individual drug research frequency (n = 138 drug mentions); the inset donut chart displays the proportional distribution of the top 10 agents. **(B)** Horizontal ranking of drug-specific research frequency with absolute counts and relative percentages. **(C)** Aggregate research frequency by therapeutic class (left) and the corresponding proportional distribution (right donut chart), with the number of distinct agents per class annotated above each bar. **(D)** Distribution of agents across research-intensity tiers.

**Figure 8:**
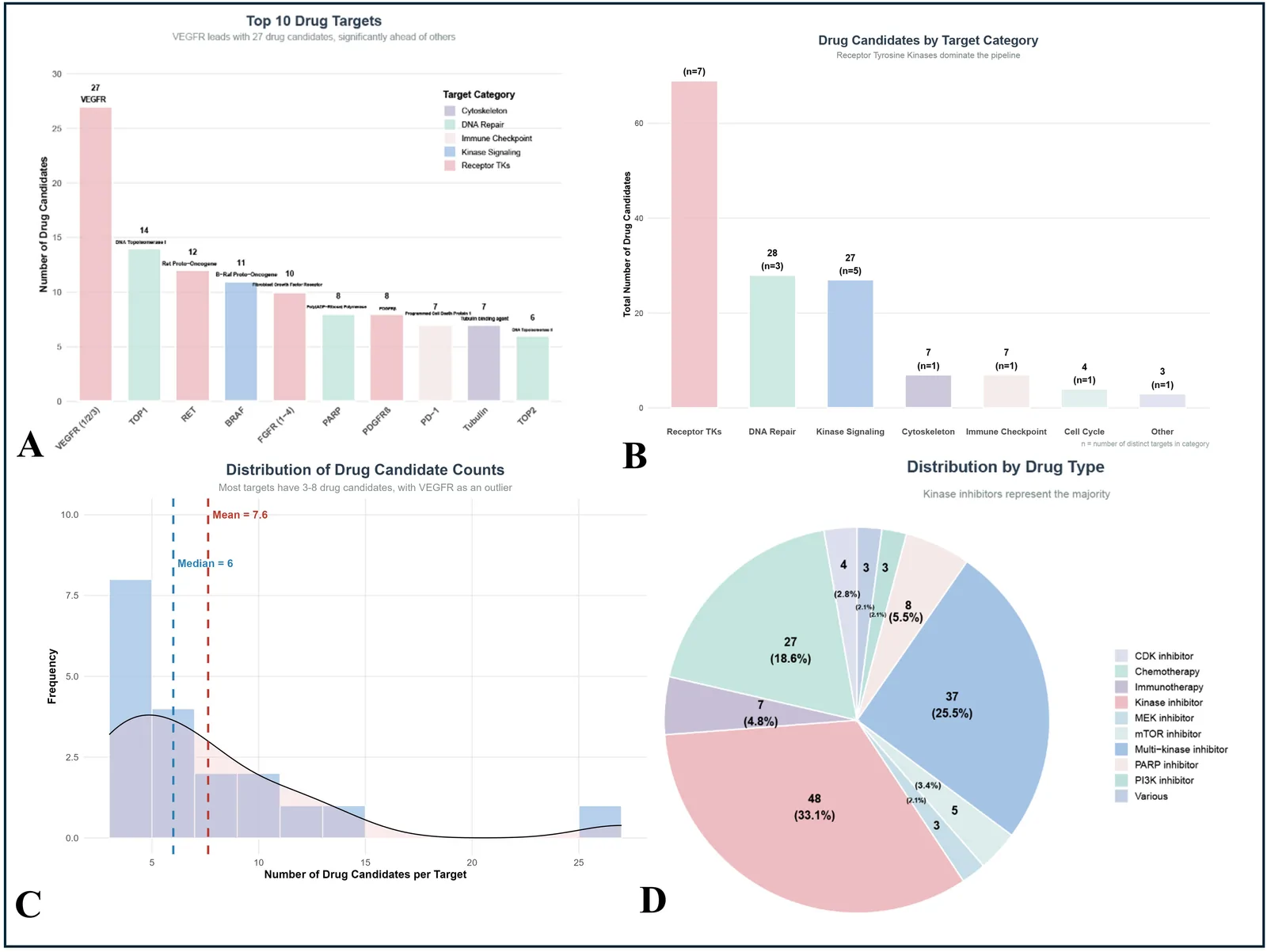
Distribution of therapeutic targets for hepatoblastoma. **(A)** Top 10 molecular targets by number of unique drug candidates. **(B)** Aggregate drug candidate counts stratified by target category. **(C)** Frequency distribution of drug candidate counts per target. **(D)** Proportional composition of the pipeline by drug modality.

Phase distribution was heavily skewed toward early-stage studies (**Table 5**). Phase I trials constituted 53.9%, Phase II 21.5%, Phase III 11.0%, and Phase IV 2.7%; combined Phase I/II or II/III trials accounted for 3.6%, and 7.3% were classified as not available or early Phase I (**Figure 6**). By intervention category, chemotherapy predominated (68.5%), followed by surgical or radiotherapy (15.1%), targeted therapy (9.1%), cellular therapy (4.6%), and immunotherapy (2.7%). Cisplatin was the most frequently recorded chemotherapeutic agent, appearing in 78 of 219 trials (35.6%). Among 138 drug mentions, sorafenib (20 mentions, 14.5%), cisplatin (19, 13.8%), and carboplatin (16, 11.6%) were the most common (**Figure 7**). Alkylating agents contributed 50 mentions (36.2%) across 4 distinct drugs, followed by antimetabolites and targeted therapy (24 mentions each, 17.4%). Shannon’s diversity index for drug classes was 1.245 (H′~max~ = 1.792; Pielou’s evenness J′ = 0.694). Analysis of 145 drug candidate–target dyads revealed VEGFR as the most targeted molecule (27 candidates), followed by DNA topoisomerase I (13), RET (11), and BRAF (10) (**Figure 8**). Receptor tyrosine kinases comprised 7 distinct targets with 67 total candidates. Kinase inhibitors constituted the largest modality (48, 33.1%), followed by multi-kinase inhibitors (37, 25.5%) and chemotherapy agents (27, 18.6%). The per-target candidate distribution was right-skewed (mean 7.6, median 6.0).

**Table 5:** Inclusion of trial characteristics at baseline.

Characteristic	Included trials (n = 219)	Excluded non-active trials (n = 32)	P-value
Phase distribution			
Phase I	53.9%	40.6%	0.18
Phase II	21.5%	18.8%	0.74
Phase III	11.0%	9.4%	0.82
Phase IV	2.7%	0%	0.59
Phase I/II or II/III	3.6%	6.3%	0.55
NA/Early Phase I	7.3%	25.0%	**0.008**
Intervention category			
Chemotherapy	68.5%	62.5%	0.52
Targeted therapy	9.1%	6.3%	0.75
Immunotherapy	2.7%	3.1%	0.92
Surgical/Radiotherapy	15.1%	21.9%	0.31
Cellular therapy	4.6%	6.3%	0.68
Sponsor type			
Academic	42.0%	59.4%	**0.048**
Industry	18.7%	9.4%	0.23
Collaborative	39.3%	31.3%	0.40
Geographic region (sponsor)			
North America	38.8%	34.4%	0.63
Europe	31.5%	37.5%	0.51
East Asia	24.2%	18.8%	0.51
Other	5.5%	9.4%	0.42
Median time from registration to status change	N/A (active/completed)	2.4 years (IQR 1.1–4.7)	—

IQR, interquartile range.

Bold values indicate statistical significance (P < 0.05).

**Figure 9:**
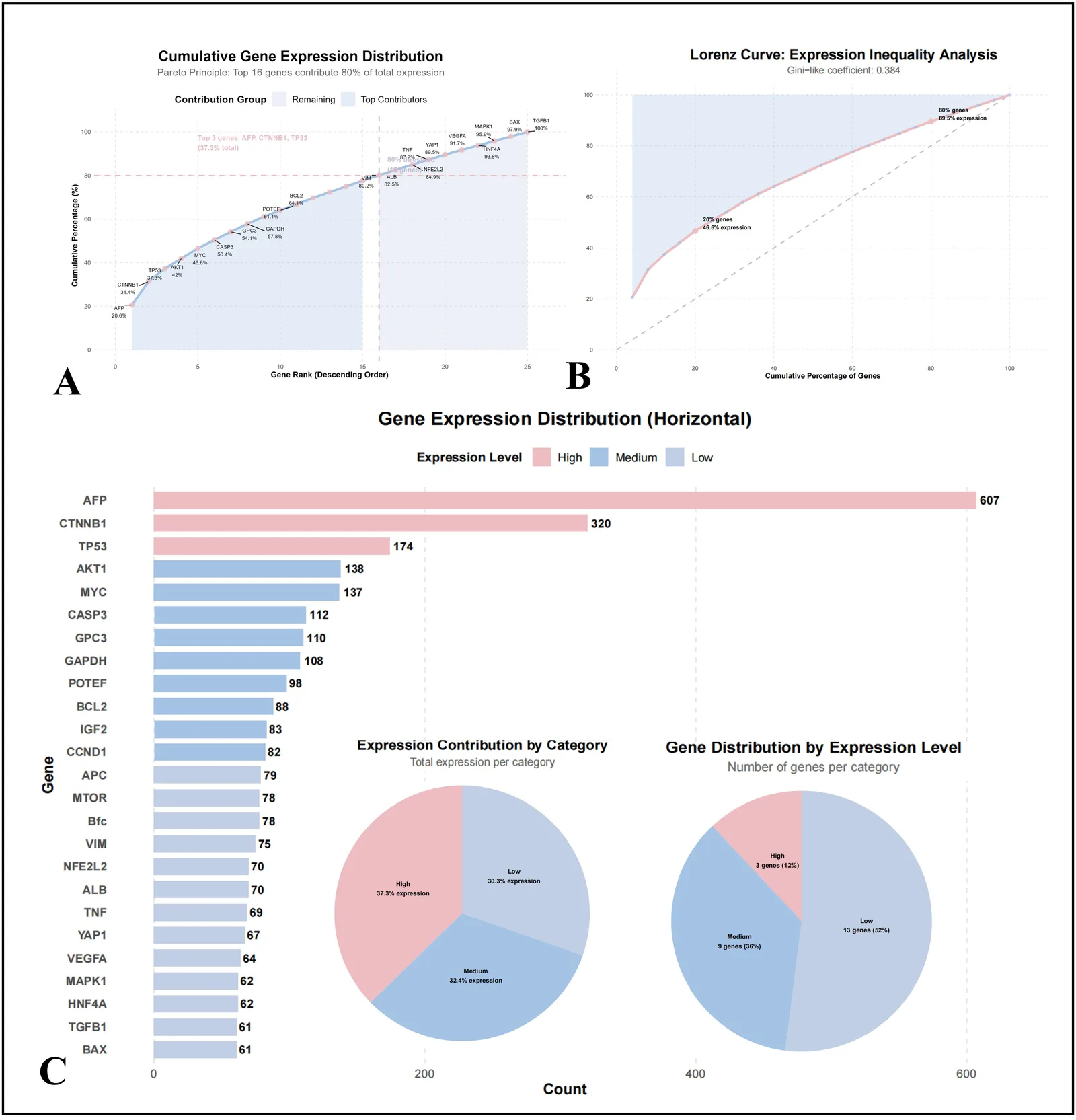
The distribution of the gene. **(A)** Pareto chart of cumulative gene mention frequency, with individual genes ranked by descending research attention. The top three genes (AFP, CTNNB1, TP53) collectively account for 37.3% of total mentions, and the top 16 genes reach the 80% cumulative threshold. **(B)** Lorenz curve illustrating the inequality of research distribution across the gene landscape, with a Gini-like coefficient of 0.384. **(C)** Horizontal bar chart of absolute mention counts for the 25 most frequently investigated genes, color-stratified by research-intensity tier: high (≥150 mentions), medium (60–149 mentions), and low (<60 mentions). Inset pie charts display the proportional contribution of each tier to total mentions (left) and the distribution of gene counts across tiers (right).

The 25 most frequently investigated genes exhibited marked concentration of research attention (**Figure 9**; **Table 6**). AFP was the most frequently mentioned (607), followed by CTNNB1 (320), TP53 (174), AKT1 (138), and MYC (137). The Lorenz curve yielded a Gini-like coefficient of 0.384; 20% of genes contributed 46.6% of cumulative mentions. High-expression genes (≥150 mentions, n = 3) represented 12% of the gene set but 37.3% of total mention volume. IGF2 was mentioned 83 times, GPC3–110 times, and YAP1–67 times.

**Table 6:** Detailed analysis of genes.

Gene	D1: pub count	D2: biological prevalence in HB	D2 source	D3: actionability status	D3 evidence
AFP	607	Serum biomarker (universal elevation)	Clinical cohorts	Diagnostic/prognostic only; not directly druggable	No targeted AFP trials
CTNNB1	320	70–90% somatic mutation (exon 3)	Sumazin et al., 2022; Euskirchen et al., 2014	Preclinical only (ICG-001, WNTinib, siRNA)	No registered HB-specific trials
TP53	174	5–10% (higher in anaplastic/chemoresistant subtypes)	Molecular profiling studies	Limited; MDM2 inhibitors not tested in HB	Preclinical
AKT1	138	Pathway activation common; focal AKT1 mutations rare	PI3K/AKT/mTOR pathway studies	Indirect (mTOR inhibitors in early-phase trials)	2 trials (everolimus, temsirolimus)
MYC	137	20–30% copy-number gain/IGF2-driven indirect activation	Genomic studies	BET inhibitors (JQ1 preclinical)	0 HB-specific trials
IGF2	83	71% overexpression	Abril-Fornaguera et al., 2023	Preclinical actionable (xentuzumab + cisplatin in PDX)	0 registered trials
GPC3	110	~80% HB expression (fetal hepatic marker)	Immunohistochemistry cohorts	Clinically investigational (CAR-T, antibody-drug conjugates)	3 registered trials (China, NCT04420432)
YAP1	67	30–40% amplification in high-risk HB	Hippo pathway studies	Preclinical (verteporfin analogs)	0 registered trials
NFE2L2	70	<5% (not established as HB driver)	Pan-cancer oxidative stress literature	None in HB	0 trials
VEGFA	64	Variable expression; angiogenic dependency	HB vascular microenvironment studies	Actively targeted (sorafenib, bevacizumab repurposed from adult HCC)	8 trials (adult agents repurposed)

## Discussion

Our integrated analysis of 219 clinical trials from 16 international registries and 2,635 PubMed-indexed publications reveals a striking paradox: hepatoblastoma is among the most molecularly deciphered pediatric solid tumors, yet two decades of intensified global research have failed to produce a single molecularly targeted therapy approved specifically for this disease. This disconnection between biological understanding and clinical implementation defines what we term the “HB translational paradox.”

Our bibliometric profiling identified CTNNB1 as the second most frequently investigated gene (n = 485 mentions), with somatic mutations documented in over 80% of patients under 8 years of age. Despite this near-universal genomic dependency, our registry audit found that no Wnt pathway antagonist has advanced beyond preclinical evaluation in registered HB trials. Preclinical studies have demonstrated therapeutic potential through siRNA knockdown, ICG-001, miRNA mimetics, and the multi-kinase inhibitor WNTinib in patient-derived xenograft and organoid models (12). This inertia cannot be attributed solely to disease rarity; infantile fibrosarcoma with TRK fusions achieved regulatory approval for larotrectinib and entrectinib through single-arm basket trials despite comparable epidemiological constraints (24, 25). Rather, our data indicate that these patterns are consistent with systemic undervaluation of pediatric drug development, though our analysis cannot establish whether this reflects funding priorities, regulatory thresholds, or commercial feasibility constraints. Although the RACE for Children Act of 2017 mandated pediatric trials for new oncology drugs with relevant molecular targets—resulting in 60% of post-2020 submissions including pediatric requirements versus none pre-implementation (23, 26) —targeted therapies and immunotherapies collectively constitute merely 9.1% of HB interventional studies in our cohort. This persistent pharmacologic conservatism underscores a disconnect between genomic knowledge and interventional design.

Our analysis further reveals that while no molecular biomarker is currently used in routine clinical practice to guide frontline HB treatment selection, the literature corpus indicates active investigation of several biomarker-therapeutic dyads. Glypican-3 (GPC3) is expressed in approximately 80% of HB tumors and represents the most advanced targeted immunotherapy approach, with a phase I trial (NCT04420432, China) and several investigator-initiated studies evaluating GPC3-directed CAR-T cells in pediatric solid tumors including HB (27–29). Serial AFP monitoring is widely employed to assess response and detect relapse, and emerging evidence supports integrating AFP decline kinetics as a stratification biomarker in risk-adapted trials (30, 31). However, standardized thresholds remain to be validated. Pilot studies have demonstrated the feasibility of circulating tumor DNA (ctDNA) detection in HB, with CTNNB1 mutations detectable in plasma and potentially informative for minimal residual disease monitoring (32). Nevertheless, our registry audit found that no prospective trial currently uses ctDNA to guide therapy, and despite the greater than 80% mutation prevalence, no trial prospectively stratifies patients by CTNNB1 status for Wnt pathway antagonist therapy. These observations illustrate that continued molecular characterization is necessary but insufficient; the imperative is to embed biomarker stratification as a mandatory inclusion criterion in prospective trials, thereby transforming biological insight into therapeutic innovation.

The most alarming finding from our registry analysis is a profound phase asymmetry: Phase I and II trials constituted 86.3% of interventional studies, whereas Phase III and IV trials accounted for only 13.7%. This represents a structural imbalance in phase distribution that may indicate a crisis of late-stage validation infrastructure, though our data cannot confirm causality or distinguish between supply-side and demand-side mechanisms. The SIOPEL-3 and COG AHEP0731 protocols established cisplatin-based chemotherapy as the cornerstone of treatment over two decades ago (33–35). Since then, no Phase III trial in our cohort has introduced a novel therapeutic class. SIOPEL-6 stands as the rare exception, demonstrating that sodium thiosulfate reduces cisplatin-induced hearing loss without compromising survival, leading to FDA approval in September 2022 (36). Yet this landmark achievement has not catalyzed subsequent late-stage infrastructure. The absence of Phase III/IV trials creates conditions consistent with a self-perpetuating cycle: without definitive efficacy data, regulators hesitate to endorse new agents, while pharmaceutical sponsors withdraw from commercially marginal indications.

Our registry analysis identified cisplatin as the most frequently utilized chemotherapeutic agent, appearing in 78 of 219 trials (35.6%) as either monotherapy or in combination. Strikingly, our cohort-level audit revealed that no registered trial specifically targeted cisplatin resistance mechanisms as a primary endpoint. This absence is particularly notable given that 86.3% of interventional studies in our dataset were Phase I/II trials—the developmental phases where combination strategies are conventionally explored. Yet our analysis demonstrates that these mechanistic insights have not been incorporated into clinical trial endpoints, revealing a persistent disconnect between molecular understanding and interventional design in the global hepatoblastoma trial landscape.

Our ecological diversity analysis reveals that the therapeutic target portfolio is concentrated in four molecular entities: VEGFR, TOP1, RET, and BRAF inhibitors—agents overwhelmingly repurposed from adult malignancies. This strategy misaligns with HB biology. For example, VEGFR inhibition targets angiogenic signaling in tumors with fetal hepatic sinusoid architecture distinct from adult hepatocellular carcinoma (37, 38). The underrepresentation of developmentally relevant targets exemplifies the consequences of adult-centric repurposing. Our target-diversity analysis revealed that the Hippo/YAP signaling axis was represented in only 1.4% of target-directed trials (3 of 219). This underrepresentation contrasts sharply with preclinical findings: YAP1 amplification has been shown to drive chemoresistance in 30–40% of high-risk hepatoblastomas through mTORC1 activation and SLC38A1-mediated amino acid transport (39, 40). The near-absence of YAP-directed trials in our cohort suggests that this biologically validated vulnerability remains outside the current clinical development paradigm for pediatric liver malignancy.

Trial registry analysis identified immune checkpoint inhibitors in only 1.8% of interventional studies (4 of 219). By comparison, these agents have transformed the therapeutic landscape of adult hepatocellular carcinoma, as demonstrated by the IMbrave150 (atezolizumab plus bevacizumab) and HIMALAYA (STRIDE regimen) trials (41). The immunogenic potential conferred by hepatoblastoma’s fetal differentiation state has been recognized in translational studies; nevertheless, our data indicate that this biological opportunity remains virtually untested in registered pediatric trials, representing a substantial translational gap.

Bibliometric profiling identified IGF2 among the top ten most frequently investigated genes (83 mentions across the literature corpus); however, our registry audit found zero registered trials targeting the IGF2 pathway. Preclinical work has established IGF2 overexpression—present in 71% of hepatoblastomas and associated with progenitor cell features and shorter recurrence-free survival—as an actionable genomic driver; the anti-IGF1/2 monoclonal antibody xentuzumab has demonstrated synergistic antitumor effects with cisplatin in patient-derived xenograft models (42, 43). Our findings thus highlight a disconnect between publication-level molecular interest and trial-level therapeutic execution.

Gini coefficient for trial distribution across nations was 0.58; quantifying geographic inequity raises the hypothesis that exclusion of high-burden regions from trial networks constitutes both ethical failure and scientific liability, a proposition testable through comparative molecular epidemiology but not verifiable within the present ecological analysis. Trial concentration in North America, Europe, and East Asia excludes sub-Saharan Africa and South Asia, where 70% of HB-related deaths occur (44, 45). Low-SDI regions accounted for 987 deaths (40.9% global HB mortality) in 2021 despite minimal research output. The correlation between trial density and healthcare infrastructure indices—specifically GDP per capita (Spearman’s ρ = 0.72, P < 0.001) and pediatric oncology workforce density (ρ = 0.68, P < 0.001)—rather than disease burden (ρ = 0.19, P = 0.31) reveals a fundamental misalignment between research investment and epidemiological priority. This exclusion has molecular consequences: mutational landscapes and environmental exposures in high-burden settings remain uncharacterized. Global analyses confirm that only 43% of childhood cancer trials include low- and middle-income countries, home to 80% of the global pediatric population, and CAR T-cell trials show a 73.3% concentration in high-income countries with zero African participation. Equitable multinational networks are epistemologically imperative—without representative global cohorts, HB molecular taxonomy remains incomplete.

Registry-reported metadata introduces potential misclassification bias regarding trial phase and target annotations, mitigated but not eliminated through dual-independent extraction and adjudication. Our bibliometric analysis captures publication volume and gene mention frequency rather than citation impact or translational outcome, and our geographic correlation analyses are ecological in nature, precluding individual-level causal inference. An important limitation of our trial landscape analysis is the exclusion of non-active registrations (n = 32), which introduces a survivorship bias toward trials that achieved sufficient momentum to remain active or complete. Our shadow analysis demonstrates that these excluded trials were predominantly early-phase, academically sponsored, and lacking formal phase designation—suggesting that attrition occurs at the feasibility stage rather than during late-phase testing.

The HB ecosystem stands at an inflection point. Two decades of molecular characterization have yielded detailed biological maps; the imperative is to build a clinical infrastructure capable of navigating them. Our analysis reveals a field robust in early-phase discovery but constrained in late-stage validation, mechanistically narrow in target selection, and structurally inequitable in global distribution. Without strategic investment in pediatric-specific preclinical platforms, biomarker-integrated trial design, and equitable multinational networks, HB will remain a paradigm of the translational gap described herein, the mechanistic roots of which await further investigation—a disease whose molecular secrets we have learned but whose clinical mysteries we have failed to solve.

## Conclusion

This multi-database analysis of 219 trials and 2,635 publications reveals a hepatoblastoma research ecosystem robust in early-phase productivity yet structurally constrained in translational impact. The marked phase skew—86.3% Phase I/II versus 13.7% Phase III/IV trials—reflects persistent validation barriers in rare pediatric oncology. Therapeutic target diversity remains mechanistically narrow (Shannon’s *H′* = 1.12), with Hippo/YAP and immune checkpoints critically underrepresented despite strong pathogenic relevance. Rectifying these deficits demands pediatric-specific preclinical platforms, mandatory multi-omics integration in prospective trials, and equitable multinational network expansion to align molecular understanding with clinical implementation across the global spectrum of this childhood malignancy.

## Data Availability

The original contributions presented in the study are included in the article/**Supplementary Material**. Further inquiries can be directed to the corresponding author.

## Glossary

AFP: alpha-fetoprotein
AKT1: AKT serine/threonine kinase 1
ALB: albumin
APC: annual percent change
ATC: Anatomical Therapeutic Chemical
BAX: BCL2-associated X, apoptosis regulator
BCL2: B-cell lymphoma 2
BIC: Bayesian Information Criterion
BRAF: B-Raf proto-oncogene, serine/threonine kinase
CAR: chimeric antigen receptor
CASP3: caspase 3
CCND1: cyclin D1
CDK: cyclin-dependent kinase
COG: Children’s Oncology Group
CTNNB1: catenin beta 1
ctDNA: circulating tumor DNA
FDA: Food and Drug Administration
FGFR: fibroblast growth factor receptor
GAPDH: glyceraldehyde-3-phosphate dehydrogenase
GPC3: glypican-3
HB: hepatoblastoma
HCC: hepatocellular carcinoma
HNF4A: hepatocyte nuclear factor 4 alpha
IGF2: insulin-like growth factor 2
IQR: interquartile range
LOESS: locally estimated scatterplot smoothing
LMIC: low- and middle-income countries
MAPK1: mitogen-activated protein kinase 1
MEK: mitogen-activated protein kinase kinase
MeSH: Medical Subject Headings
mTOR: mechanistic target of rapamycin
mTORC1: mechanistic target of rapamycin complex 1
PARP: poly (ADP-ribose) polymerase
PD-1: programmed cell death protein 1
PDGFRB: platelet-derived growth factor receptor beta
PI3K: phosphoinositide 3-kinase
POTEF: POTE ankyrin domain family member F
RACE: Research to Accelerate Cures and Equity
RET: rearranged during transfection proto-oncogene
SDI: Socio-demographic Index
SIOPEL: International Society of Paediatric Oncology
SLC38A1: solute carrier family 38 member 1
TGFB1: transforming growth factor beta 1
TKI: tyrosine kinase inhibitor
TNF: tumor necrosis factor
TOP1: DNA topoisomerase I
TP53: tumor protein p53
TRK: tropomyosin receptor kinase
VEGFA: vascular endothelial growth factor A
VEGFR: vascular endothelial growth factor receptor
VIM: vimentin
Wnt: Wingless-related integration site
YAP: Yes-associated protein.
