# Supplementary material for: Bibliometric and clinical trial landscape of hepatoblastoma (2000–2024): a multi-database analysis of global research
Source: Front Oncol. 2026 Sep 3;16:1890288. doi: 10.3389/fonc.2026.1890288 (PMC13581518; doi:10.3389/fonc.2026.1890288)
Supplement: Supplementary file 1 [file DataSheet1.docx]

**Supplementary Figure S1**

**Supplementary _Table_ S1:** PubMed literature search strategy, query syntax, and filter parameters

|  | Search # | Query | Details / Rationale |  |
| --- | --- | --- | --- | --- |
| —January first, 2025 | #1 | hepatoblastoma[MeSH Terms] | MeSH term for the primary disease. | |
|  | #2 | hepatoblastoma[Title/Abstract] | Free-text term for the primary disease. | |
|  | #3 | hepatoblastoma cancer[Title/Abstract] | Free-text variant. |  |
|  | #4 | pediatric liver tumor[Title/Abstract] | Free-text (US spelling). | |
|  | #5 | pediatric liver tumours[Title/Abstract] | Free-text (UK spelling). | |
|  | #6 | pediatric liver malignancy[Title/Abstract] | Free-text singular. |  |
|  | #7 | pediatric liver malignancies[Title/Abstract] | Free-text plural. |  |
|  | #8 | childhood liver tumor[Title/Abstract] | Free-text (US spelling). | |
|  | #9 | childhood liver tumour[Title/Abstract] | Free-text (UK spelling). | |
|  | #10 | childhood liver malignancy[Title/Abstract] | Free-text singular. |  |
|  | #11 | childhood liver malignancies[Title/Abstract] | Free-text plural. |  |
|  | #12 | infantile liver cancer[Title/Abstract] | Free-text for infant population. | |
|  | #13 | infantile liver tumour[Title/Abstract] | Free-text (UK spelling). | |
|  | #14 | liver neoplasms[MeSH Terms] AND "child"[MeSH Terms] | MeSH combination for liver cancer in children (ages 1–12 years). | |
|  | #15 | liver neoplasms[MeSH Terms] AND "infant"[MeSH Terms] | MeSH combination for liver cancer in infants (ages 0–12 months). | |
|  | #16 | #1 OR #2 OR #3 OR #4 OR #5 OR #6 OR #7 OR #8 OR #9 OR #10 OR #11 OR #12 OR #13 OR #14 OR #15 | Population/Intervention (P/I) block – combines all disease and age-related concepts. | |
|  | #17 | ("2000/01/01"[Date - Publication] : "2025/01/01"[Date - Publication]) | Date filter – restricts to studies published from January 1, 2000, to January 1, 2025. | |
|  | #18 | humans[MeSH Terms] | Species filter – excludes animal studies. | |
|  | #19 | english[Language] | Language filter – restricts to publications in English. | |
|  | #20 | #16 AND #17 AND #18 AND #19 | Final combination – applies all filters to the primary search block. | |
|  | Hits retrieved: 8,963 | | | |

**Supplementary Table S2:** Search parameters and initial yield across 16 international clinical trial registries

| Registry | Search Interface | Search Syntax / Parameters | Hits Retrieved |
| --- | --- | --- | --- |
| ClinicalTrials.gov | Advanced Search | Condition or disease: hepatoblastoma; Study type: Interventional studies; Status: All (active/completed); Age: Child (birth–17) | 68 |
| EU Clinical Trials Register | Search for Trials | Full text search: hepatoblastoma; Age group: Children; Trial status: All | 35 |
| Chinese Clinical Trial Registry (ChiCTR) | Basic Search | Keyword: 肝母细胞瘤 OR hepatoblastoma; Study type: 干预性研究 | 28 |
| China Drug Clinical Trial Registration | Disease Search | Keyword: 肝母细胞瘤 | 12 |
| ISRCTN | Standard Search | Condition: hepatoblastoma; Age range: 0–18 years | 8 |
| JRCTR (Japan) | Trial Search | Disease name: 肝芽腫 OR hepatoblastoma; Age: 新生児・乳児・小児 | 6 |
| UMIN-CTR (Japan) | Search | Disease: hepatoblastoma | 4 |
| ANZCTR | Basic Search | Condition: hepatoblastoma; Age: Children | 5 |
| CTRI (India) | Search Trials | Condition: hepatoblastoma | 3 |
| KCT (Korea) | Search | Disease: hepatoblastoma | 2 |
| ReBEC (Brazil) | Search | Condition: hepatoblastoma | 2 |
| TCTR (Thailand) | Search | Condition: hepatoblastoma | 1 |
| PACTR (Africa) | Search | Condition: hepatoblastoma | 1 |
| SLCTR (Sri Lanka) | Search | Condition: hepatoblastoma | 0 |
| IRCT (Iran) | Search | Condition: hepatoblastoma | 1 |
| NTR (Netherlands) | Search | Condition: hepatoblastoma | 2 |

_Total initial records identified: 317 (after removal of obvious non-interventional records at the interface level)._

**Supplementary Table S3.** Characteristics and registry metadata of excluded non-active or incomplete hepatoblastoma trials

|  | **_No._** | **_Registration Number_** | **_Date_** | **_Country/Region_** | **_Study Type_** | **_Trial Phase_** |
| --- | --- | --- | --- | --- | --- | --- |
| 1 | 1 | NCT03739827 | 2018 | United States | Observation | NA |
| 2 | 16 | NCT06541652 | 2024 | France | Observation | NA |
| 3 | 17 | NCT06822842 | 2025 | United States | Observation | NA |
| 4 | 18 | NCT03959800 | 2019 | United States | Observation | NA |
| 5 | 20 | NCT03382158 | 2017 | United States | Observation | NA |
| 6 | 28 | NCT05556642 | 2022 | United States | Observation | NA |
| 7 | 31 | NCT06230328 | 2024 | Brazil | Observation | NA |
| 8 | 36 | NCT04455698 | 2020 | United States | Interventional | NA |
| 9 | 39 | NCT06443632 | 2024 | China | Interventional | NA |
| 10 | 42 | NCT04315883 | 2020 | United States | Observation | NA |
| 11 | 43 | NCT05245123 | 2022 | Germany | Observation | NA |
| 12 | 44 | NCT04181138 | 2019 | Canada, United States | Observation | NA |
| 13 | 47 | NCT05763173 | 2023 | China | Observation | NA |
| 14 | 50 | NCT05064306 | 2021 | United States | NA | NA |
| 15 | 51 | NCT06190574 | 2024 | China | Observation | NA |
| 16 | 53 | NCT01505569 | 2012 | United States | Interventional | NA |
| 17 | 54 | NCT05756660 | 2023 | United States | Interventional | NA |
| 18 | 58 | NCT05400031 | 2022 | Turkey | Interventional | NA |
| 19 | 59 | NCT06045949 | 2023 | China | Observation | NA |
| 20 | 64 | NCT05693519 | 2023 | Netherlands | Observation | NA |
| 21 | 66 | NCT02557750 | 2015 | Egypt | Observation | NA |
| 22 | 68 | NCT05170282 | 2021 | China | Observation | NA |
| 23 | 70 | NCT05076071 | 2021 | China | Observation | NA |
| 24 | 71 | NCT04132882 | 2019 | Turkey | NA | NA |
| 25 | 72 | NCT03645655 | 2018 | China | Observation | NA |
| 26 | 73 | NCT02425397 | 2015 | France | Observation | NA |
| 27 | 87 | NCT03913416 | 2019 | France | Interventional | NA |
| 28 | 90 | NCT04012021 | 2019 | Italy | Interventional | NA |
| 29 | 102 | NCT01336881 | 2011 | United States | Observation | NA |
| 30 | 107 | NCT00624962 | 2008 | — | Interventional | NA |
| 31 | 110 | NCT00228683 | 2005 | United States | Interventional | NA |
| 32 | 114 | NCT01533779 | 2012 | Israel | Observation | NA |
| 33 | 176 | UMIN000056899 | 2025 | Japan | Observation | NA |
| 34 | 179 | jRCT1060240085 | 2024 | Japan | Observation | NA |
| 35 | 180 | UMIN000055367 | 2024 | Japan | Observation | NA |
| 36 | 181 | UMIN000054107 | 2024 | Japan | Interventional | NA |
| 37 | 184 | UMIN000022324 | 2016 | Japan | Interventional | NA |
| 38 | 185 | UMIN000021931 | 2016 | Japan | Interventional | NA |
| 39 | 194 | UMIN000026474 | 2017 | Japan | Interventional | NA |
| 40 | 197 | ISRCTN17869351 | 2001 | Austria, Belgium, Czech Republic | Interventional | NA |
| 41 | 198 | ISRCTN87675276 | 2016 | England, United Kingdom | Interventional | NA |
| 42 | 199 | ISRCTN16342840 | 2011 | Brazil | Interventional | NA |
| 43 | 200 | ISRCTN64076941 | 2024 | Spain | Interventional | NA |
| 44 | 202 | CTRI/2024/06/069360 | 2024 | India | Observation | NA |
| 45 | 203 | CTRI/2023/03/051221 | 2023 | India | Observation | NA |
| 46 | 204 | CTRI/2022/07/044087 | 2022 | India | Interventional | NA |
| 47 | 205 | CTRI/2021/03/032430 | 2021 | India | Interventional | NA |
| 48 | 207 | CTRI/2021/05/033611 | 2021 | India | Observation | NA |
| 49 | 209 | DRKS00007623 | 2014 | Austria, Belgium, Czechia | Observation | NA |
| 50 | 210 | DRKS00027082 | 2021 | Germany | Observation | NA |
| 51 | 211 | DRKS00024485 | 2022 | Germany | Observation | NA |
| 52 | 212 | DRKS00023554 | 2021 | Germany | Observation | NA |
| 53 | 213 | DRKS00010427 | 2016 | Germany | Interventional | NA |
| 54 | 214 | TCTR20220418005 | 2022 | India | Observation | NA |
| 55 | 215 | TCTR20210221001 | 2021 | Thailand | Observation | NA |
| 56 | 216 | 219451 | 2024 | Argentina, Czechia, Spain | Interventional | NA |

**Supplementary Table S4.** Sensitivity analysis of geographic inequality metrics under sponsor-based versus recruitment-based attribution

| **Metric** | **Sponsor-Based (Original)** | **Recruitment-Based (Revised)** | **Qualitative Impact** |
| --- | --- | --- | --- |
| **Top 3 countries by trial count** | USA (45), China (38), Germany (22) | USA (52), China (41), Germany (28) | Rank order unchanged; absolute counts increase for multinational sponsors |
| **Trials originating from sub-Saharan Africa** | 2 | 4 | Slightly higher when recruitment sites in South Africa/Nigeria are counted |
| **Gini coefficient (trial distribution across nations)** | 0.58 | 0.54 | Marginally lower under recruitment-based attribution, but still indicates **substantial inequality** (>0.40 threshold) |
| **Spearman ρ (trial density vs. GDP per capita)** | 0.72 (P < 0.001) | 0.68 (P < 0.001) | Slightly attenuated but remains **strongly significant** |
| **Spearman ρ (trial density vs. pediatric oncology workforce)** | 0.68 (P < 0.001) | 0.64 (P < 0.001) | Same pattern |
| **Spearman ρ (trial density vs. disease burden)** | 0.19 (P = 0.31) | 0.22 (P = 0.24) | Remains **non-significant** |
| **% of trials with any LMIC recruitment** | Not calculated originally | **43.8%** | New finding: even under recruitment-based counting, <50% of trials reach LMICs |

**Supplementary Table S5.** Operational definitions and analytical units for three-dimensional gene-level bibliometric profiling

| **Dimension** | **Definition** | **Examples** | **Analytical Unit** |
| --- | --- | --- | --- |
| **D1: Bibliometric Attention** | Frequency of mention in the 2,953 included publications | AFP (n = 607), CTNNB1 (n = 320) | Raw publication count; keyword extraction from titles/abstracts/full-text |
| **D2: Biological Prevalence** | Somatic mutation, copy-number alteration, or epigenetic dysregulation frequency in HB tumor cohorts | CTNNB1 (70–90%), NFE2L2 (rare in HB; see below), IGF2 (71%) | Population-based genomic studies; TCGA/Pediatric Cancer Genome Project data |
| **D3: Therapeutic Actionability** | Current or investigational targeting in clinical trials or preclinical pipelines | VEGFR (28 trials), GPC3 (CAR-T trials), CTNNB1 (preclinical only) | Trial registry annotation; drug-target database cross-reference (DrugBank, Open Targets) |

**Supplementary Table S6**. Independent audit and final reconciliation of key statistical metrics

| **Statistic** | **Original Report** | **Reviewer Citation** | **Corrected (Verified)** | **Location in Revised MS** |
| --- | --- | --- | --- | --- |
| GDP per capita vs. trial density (Spearman ρ) | 0.72 | 0.81 | **0.69 (95% CI 0.52–0.81)** | Results; Supplementary Table S14 |
| Pediatric oncology workforce vs. trial density (Spearman ρ) | 0.68 | 0.54 | **0.66 (95% CI 0.48–0.79)** | Results; Supplementary Table S14 |
| Gini coefficient (trial distribution across nations) | 0.58 | 0.76 | **0.58 (bootstrap 95% CI 0.51–0.65)** | Results; Supplementary Table S15 |
| Shannon diversity index (therapeutic targets, 4 categories) | Not explicitly reported | 2.42 | **H′ = 1.078; J′ = 0.778** | Results; Supplementary Table S11 |
| Shannon diversity index (drug classes, 6 categories) | Not reported | 1.86 | **H′ = 1.245; J′ = 0.694** | Results; Supplementary Table S16 |

**Supplementary Table S7**. Country-level trial distribution, healthcare infrastructure indices, and hepatoblastoma disease burden metrics

| **Country** | **Trials (Sponsor-Based)** | **Trials (Recruitment-Based)** | **GDP per Capita (USD, 2023)** | **Pediatric Oncology Workforce (per million children)** | **Age-Standardized HB Mortality (per 100,000)** | **HB Incidence (per million children)** |
| --- | --- | --- | --- | --- | --- | --- |
| United States | 45 | 52 | 76,398 | 12.4 | 0.08 | 1.2 |
| China | 38 | 41 | 12,614 | 2.1 | 0.42 | 1.8 |
| Germany | 22 | 28 | 51,384 | 8.7 | 0.06 | 0.9 |
| France | 18 | 24 | 46,320 | 7.9 | 0.05 | 0.8 |
| United Kingdom | 12 | 19 | 48,890 | 9.2 | 0.06 | 1.0 |
| Japan | 8 | 10 | 33,834 | 6.3 | 0.04 | 0.7 |
| Canada | 7 | 9 | 53,247 | 10.1 | 0.07 | 1.1 |
| Australia | 5 | 8 | 63,487 | 11.3 | 0.06 | 0.9 |
| Italy | 5 | 7 | 37,700 | 5.8 | 0.05 | 0.8 |
| South Korea | 4 | 5 | 33,592 | 4.2 | 0.05 | 0.8 |
| India | 1 | 4 | 2,389 | 0.3 | 0.38 | 1.5 |
| Brazil | 2 | 5 | 8,917 | 0.9 | 0.22 | 1.3 |
| South Africa | 0 | 3 | 6,190 | 0.7 | 0.28 | 1.4 |

Missing data: GDP per capita and pediatric oncology workforce data were unavailable for 3 countries (Iran, Sri Lanka, North Korea). These countries were excluded from the corresponding Spearman correlations (pairwise deletion), reducing the effective n from 28 to 25 for these analyses. This is now explicitly stated.

Data sources:

GDP per capita: World Bank Open Data (2023 estimates)

Pediatric oncology workforce: SIOP-ICCD Global Mapping of Pediatric Oncology Workforce (2022 report) and national health workforce censuses

HB mortality and incidence: GBD 2021 (IHME, 2024 release)

**Supplementary Table S8**. Shannon diversity index and Pielou's evenness for drug classes (6-class model)

| **Drug Class** | **Trials Mentioning** | **Proportion (p_i)** | **p_i × ln(p_i)** |
| --- | --- | --- | --- |
| Platinum agents | 46 | 0.333 | −0.366 |
| Topoisomerase inhibitors | 26 | 0.188 | −0.315 |
| Antimetabolites | 24 | 0.174 | −0.295 |
| Targeted therapy (non-platinum, non-TKI) | 24 | 0.174 | −0.295 |
| Microtubule inhibitors | 10 | 0.072 | −0.189 |
| Chemoprotectants / Others | 8 | 0.058 | −0.163 |
| **Total** | **138 mentions** | **0.999** | **H′ = 1.245** |

Note: The unit here is drug mentions (a trial may contribute to multiple categories). H′_max = ln(6) = 1.792; J′ = 1.245 / 1.792 = 0.694.

**Supplementary Table S9**. Bayesian Information Criterion–based model selection for joinpoint regression of temporal trends

| **Category** | **Model (Joinpoints)** | **BIC** | **Permutation Test P-value** | **Selected?** |
| --- | --- | --- | --- | --- |
| **Publication** | 0 joinpoints | 187.4 | — | No |
|  | 1 joinpoint | 171.2 | 0.003 | No |
|  | **2 joinpoints** | **158.6** | **0.021** | **Yes** |
|  | 3 joinpoints | 156.3 | 0.18 | No (not significantly better than 2) |
| **Clinical trials** | 0 joinpoints | 142.1 | — | No |
|  | 1 joinpoint | 128.7 | 0.008 | No |
|  | **2 joinpoints** | **119.3** | **0.015** | **Yes** |

**Supplementary Table S10**. Segment-specific annual percent change estimates with 95% confidence intervals from joinpoint regression

| **Category** | **Segment** | **Years** | **APC (%)** | **95% CI** | **Standard Error** | **P-value** | **Interpretation** |
| --- | --- | --- | --- | --- | --- | --- | --- |
| **Publication** | 1 | 2005–2012 | +4.2 | +1.8 to +6.7 | 1.25 | 0.002 | Steady growth |
|  | 2 | 2012–2017 | −1.1 | −4.3 to +2.2 | 1.58 | 0.48 | Plateau (non-significant) |
|  | 3 | 2017–2024 | +11.8 | +7.9 to +15.8 | 1.95 | <0.001 | Accelerated growth |
| **Clinical trials** | 1 | 2001–2012 | +18.4 | +11.2 to +25.9 | 3.72 | <0.001 | — |
|  | 2 | 2012–2016 | +4.1 | −8.7 to +18.6 | 6.98 | 0.42 | — |
|  | 3 | 2016–2024 | +8.7 | +3.2 to +14.4 | 2.78 | 0.004 | — |
