# Supplementary material for: Bibliometric and clinical trial landscape of hepatoblastoma (2000–2024): a multi-database analysis of global research
Source: Front Oncol. 2026 Sep 3;16:1890288. doi: 10.3389/fonc.2026.1890288 (PMC13581518; doi:10.3389/fonc.2026.1890288)
Supplement: Supplementary file 2 [file Table1.docx]

Supplementary Dataset S1: Specific characteristics of the inclusion in clinical trials

| **_No._** | **_Registration Number_** | **_Date_** | **_Country/Region_** | **_Study Type_** | **_Trial Phase_** | **_Target(s)_** | **_Population_** | **_Age Range_** |
| --- | --- | --- | --- | --- | --- | --- | --- | --- |
| _1_ | _NCT03739827_ | _2018_ | _United States_ | _Observation_ | _NA_ | _NA_ | _Both_ | _4 Weeks - No limit_ |
| _2_ | _NCT03533582_ | _2018_ | _Australia, New Zealand, Puerto Rico_ | _Interventional_ | _Phase 3 Clinical_ | _1: FLT3, 2: c-Kit, 3: PDGFR, 4: PDGFRβ, 5: RET, 6: RAF1, 7: BRAF, 8: RAF, 9: TOP1, 10: TOP2, 11: VEGFR, 12: VEGFR1, 13: VEGFR2, 14: VEGFR3_ | _Both_ | _No limit - 30 Years_ |
| _3_ | _NCT03210714_ | _2017_ | _United States, Puerto Rico_ | _Interventional_ | _Phase 2 Clinical_ | _1: FGFR1, 2: FGFR2, 3: FGFR3, 4: FGFR4_ | _Both_ | _12 Months - 21 Years_ |
| _4_ | _NCT01804634_ | _2013_ | _United States_ | _Interventional_ | _Phase 2 Clinical_ | _1: CaN, 2: POLA, 3: PRIM, 4: RNR_ | _Both_ | _1 Year - 50 Years_ |
| _5_ | _NCT04928677_ | _2021_ | _United States_ | _Interventional_ | _Phase 1 Clinical_ | _1: GPC3_ | _Both_ | _1 Year - 21 Years_ |
| _6_ | _NCT03155620_ | _2017_ | _Puerto Rico, United States, Canada_ | _Interventional_ | _Phase 2 Clinical_ | _1: ALK, 2: EZH2, 3: FGFR1, 4: FGFR2, 5: FGFR3, 6: FGFR4, 7: HKMT, 8: MEK1, 9: MEK2, 10: PARP, 11: PI3K, 12: PARP1, 13: PARP2, 14: PARP3, 15: BRAF, 16: ROS1, 17: TRK, 18: MTOR_ | _Both_ | _12 Months - 21 Years_ |
| _7_ | _NCT04320888_ | _2020_ | _United States, Puerto Rico, Australia_ | _Interventional_ | _Phase 2 Clinical_ | _1: RET_ | _Both_ | _12 Months - 21 Years_ |
| _8_ | _NCT03213652_ | _2017_ | _Puerto Rico, United States, Canada_ | _Interventional_ | _Phase 2 Clinical_ | _1: ALK, 2: ROS1_ | _Both_ | _12 Months - 21 Years_ |
| _9_ | _NCT03213704_ | _2017_ | _United States, Puerto Rico_ | _Interventional_ | _Phase 2 Clinical_ | _1: TRK_ | _Both_ | _12 Months - 21 Years_ |
| _10_ | _NCT06521567_ | _2024_ | _United States_ | _Interventional_ | _Phase I/II_ | _1: TIM-3, 2: PD-1_ | _Both_ | _0 Years - 21 Years_ |
| _11_ | _NCT04195555_ | _2019_ | _United States, Puerto Rico, Australia_ | _Interventional_ | _Phase 2 Clinical_ | _1: IDH1_ | _Both_ | _12 Months - 21 Years_ |
| _12_ | _NCT03698994_ | _2018_ | _United States, Puerto Rico_ | _Interventional_ | _Phase 2 Clinical_ | _1: ERK1, 2: ERK2_ | _Both_ | _12 Months - 21 Years_ |
| _13_ | _NCT06047977_ | _2023_ | _United States_ | _Interventional_ | _Phase 1 Clinical_ | _1: POLA, 2: PRIM, 3: RNR_ | _Both_ | _1 Year - 21 Years_ |
| _14_ | _NCT04901702_ | _2021_ | _United States, Canada_ | _Interventional_ | _Phase I/II_ | _1: PARP1, 2: PARP2, 3: TOP1_ | _Both_ | _12 Months - 30 Years_ |
| _15_ | _NCT03654716_ | _2018_ | _United States_ | _Interventional_ | _Phase 1 Clinical_ | _1: POL, 2: MDM2, 3: MDM4, 4: RNAP, 5: p53_ | _Both_ | _1 Year - 21 Years_ |
| _16_ | _NCT06541652_ | _2024_ | _France_ | _Observation_ | _NA_ | _NA_ | _Both_ | _18 Years - No limit_ |
| _17_ | _NCT06822842_ | _2025_ | _United States_ | _Observation_ | _NA_ | _NA_ | _Both_ | _0 Years - 18 Years_ |
| _18_ | _NCT03959800_ | _2019_ | _United States_ | _Observation_ | _NA_ | _NA_ | _Both_ | _0 Years - 99 Years_ |
| _19_ | _NCT00980460_ | _2009_ | _Canada, Brazil, United States_ | _Interventional_ | _Phase 3 Clinical_ | _NA_ | _Both_ | _No limit - 21 Years_ |
| _20_ | _NCT03382158_ | _2017_ | _United States_ | _Observation_ | _NA_ | _1: Tubulin_ | _Both_ | _0 Minutes - 100 Years_ |
| _21_ | _NCT03213678_ | _2017_ | _Puerto Rico, United States_ | _Interventional_ | _Phase 2 Clinical_ | _1: PI3K, 2: MTOR_ | _Both_ | _12 Months - 21 Years_ |
| _22_ | _NCT03220035_ | _2017_ | _Puerto Rico, United States_ | _Interventional_ | _Phase 2 Clinical_ | _1: BRAF_ | _Both_ | _12 Months - 21 Years_ |
| _23_ | _NCT03213665_ | _2017_ | _Puerto Rico, United States_ | _Interventional_ | _Phase 2 Clinical_ | _1: EZH2, 2: HKMT_ | _Both_ | _12 Months - 21 Years_ |
| _24_ | _NCT02867592_ | _2016_ | _United States_ | _Interventional_ | _Phase 2 Clinical_ | _1: AXL, 2: FLT3, 3: c-Met, 4: c-Kit, 5: c-Met, 6: Mer, 7: RET, 8: TEK, 9: NTRK2, 10: TYRO3, 11: VEGFR, 13: VEGFR2, 14: VEGFR3_ | _Both_ | _2 Years - 30 Years_ |
| _25_ | _NCT05450562_ | _2022_ | _Singapore, Korea, China_ | _Interventional_ | _Phase IV_ | _1: GPC3, 2: PD-L1, 3: TNF_ | _Both_ | _18 Years - No limit_ |
| _26_ | _NCT06198296_ | _2024_ | _United States_ | _Interventional_ | _Phase 1 Clinical_ | _NA_ | _Both_ | _No limit - 21 Years_ |
| _27_ | _NCT05302921_ | _2022_ | _United States_ | _Interventional_ | _Phase 2 Clinical_ | _1: CTLA4, 2: PD-1_ | _Both_ | _1 Year - 39 Years_ |
| _28_ | _NCT05556642_ | _2022_ | _United States_ | _Observation_ | _NA_ | _NA_ | _Both_ | _No limit_ |
| _29_ | _NCT03233204_ | _2017_ | _Puerto Rico, United States_ | _Interventional_ | _Phase 2 Clinical_ | _1: PARP, 2: PARP1, 3: PARP2, 4: PARP3_ | _Both_ | _12 Months - 21 Years_ |
| _30_ | _NCT04483778_ | _2020_ | _United States_ | _Interventional_ | _Phase 1 Clinical_ | _1: CD19, 2: CD276, 3: POLA, 4: PRIM, 5: PD-1, 6: RNR_ | _Both_ | _0 Years - 26 Years_ |
| _31_ | _NCT06230328_ | _2024_ | _Brazil_ | _Observation_ | _NA_ | _NA_ | _Both_ | _18 Years - 100 Years_ |
| _32_ | _NCT04337177_ | _2020_ | _United States_ | _Interventional_ | _Phase 1 Clinical_ | _1: TOP1_ | _Both_ | _1 Year - 30 Years_ |
| _33_ | _NCT04485416_ | _2020_ | _United States_ | _Interventional_ | _Phase 1 Clinical_ | _1: TPOR_ | _Both_ | _1 Year - 18 Years_ |
| _34_ | _NCT03526250_ | _2018_ | _Puerto Rico, United States_ | _Interventional_ | _Phase 2 Clinical_ | _1: CDK4, 2: CDK6, 3: RB1_ | _Both_ | _12 Months - 21 Years_ |
| _35_ | _NCT04851119_ | _2021_ | _United States_ | _Interventional_ | _Phase I/II_ | _1: CTNNB, 2: TBL1XR1_ | _Both_ | _12 Months - 30 Years_ |
| _36_ | _NCT04455698_ | _2020_ | _United States_ | _Interventional_ | _NA_ | _NA_ | _Both_ | _18 Years - No limit_ |
| _37_ | _NCT04284774_ | _2020_ | _Puerto Rico, United States_ | _Interventional_ | _Phase 2 Clinical_ | _NA_ | _Both_ | _12 Months - 21 Years_ |
| _38_ | _NCT06638931_ | _2024_ | _Brazil_ | _Interventional_ | _Phase 2 Clinical_ | _NA_ | _Both_ | _18 Years - No limit_ |
| _39_ | _NCT06443632_ | _2024_ | _China_ | _Interventional_ | _NA_ | _NA_ | _Both_ | _No limit - 4 Years_ |
| _40_ | _NCT06235125_ | _2024_ | _United States_ | _Interventional_ | _Phase 1 Clinical_ | _1: FR_ | _Both_ | _6 Years - 17 Years_ |
| _41_ | _NCT04897321_ | _2021_ | _United States_ | _Interventional_ | _Phase 1 Clinical_ | _1: POLA, 2: PRIM, 3: RNR_ | _Both_ | _No limit - 21 Years_ |
| _42_ | _NCT04315883_ | _2020_ | _United States_ | _Observation_ | _NA_ | _NA_ | _Both_ | _0 Years - 21 Years_ |
| _43_ | _NCT05245123_ | _2022_ | _Germany_ | _Observation_ | _NA_ | _NA_ | _Both_ | _1 Month - 17 Years_ |
| _44_ | _NCT04181138_ | _2019_ | _Canada, United States_ | _Observation_ | _NA_ | _NA_ | _Both_ | _2 Years - 25 Years_ |
| _45_ | _NCT03017326_ | _2017_ | _Norway, Switzerland, Finland_ | _Interventional_ | _Phase 3 Clinical_ | _1: FLT3, 2: c-Kit, 3: PDGFR, 4: PDGFRβ, 5: RET, 6: RAF1, 7: BRAF, 8: RAF, 9: TOP1, 10: TOP2, 11: Tubulin, 12: VEGFR, 13: VEGFR1, 14: VEGFR2, 15: VEGFR3_ | _Both_ | _No limit - 30 Years_ |
| _46_ | _NCT02085148_ | _2014_ | _Spain, United Kingdom, Italy_ | _Interventional_ | _Phase 1 Clinical_ | _1: CASP3, 2: CASP9, 3: FGFR, 4: c-Kit, 5: PDGFRβ, 6: RET, 7: RAF1, 8: BRAF, 9: TIE, 10: TEK, 11: TOP1, 12: VEGFR1, 13: VEGFR2, 14: VEGFR3_ | _Both_ | _6 Months - 18 Years_ |
| _47_ | _NCT05763173_ | _2023_ | _China_ | _Observation_ | _NA_ | _NA_ | _Both_ | _No limit - 14 Years_ |
| _48_ | _NCT03618381_ | _2018_ | _United States_ | _Interventional_ | _Phase 1 Clinical_ | _1: CD19, 2: EGFR_ | _Both_ | _1 Year - 30 Years_ |
| _49_ | _NCT05608148_ | _2022_ | _Japan_ | _Interventional_ | _Phase 1 Clinical_ | _1: GCSFR, 2: IL-2_ | _Both_ | _1 Year - 24 Years_ |
| _50_ | _NCT05064306_ | _2021_ | _United States_ | _NA_ | _NA_ | _1: CD276_ | _Both_ | _No limit_ |
| _51_ | _NCT06190574_ | _2024_ | _China_ | _Observation_ | _NA_ | _NA_ | _Both_ | _No limit - 18 Years_ |
| _52_ | _NCT04634357_ | _2020_ | _United States_ | _Interventional_ | _Phase I/II_ | _1: AFP_ | _Both_ | _1 Year - 21 Years_ |
| _53_ | _NCT01505569_ | _2012_ | _United States_ | _Interventional_ | _NA_ | _1: TOP2_ | _Both_ | _No limit - 70 Years_ |
| _54_ | _NCT05756660_ | _2023_ | _United States_ | _Interventional_ | _NA_ | _NA_ | _Both_ | _1 Month - 39 Years_ |
| _55_ | _NCT04478292_ | _2020_ | _China_ | _Interventional_ | _Phase 3 Clinical_ | _NA_ | _Both_ | _No limit - 18 Years_ |
| _56_ | _NCT00716976_ | _2008_ | _United States, Australia, Canada_ | _Observation_ | _Phase 3 Clinical_ | _NA_ | _Both_ | _1 Year - 18 Years_ |
| _57_ | _NCT04308330_ | _2020_ | _United States_ | _Interventional_ | _Phase 1 Clinical_ | _1: HDAC, 2: HDAC1, 3: HDAC2, 4: HDAC3, 5: HDAC6, 6: TOP1_ | _Both_ | _1 Year - 30 Years_ |
| _58_ | _NCT05400031_ | _2022_ | _Turkey_ | _Interventional_ | _NA_ | _NA_ | _Both_ | _2 Years - 18 Years_ |
| _59_ | _NCT06045949_ | _2023_ | _China_ | _Observation_ | _NA_ | _NA_ | _Both_ | _0 Years - 18 Years_ |
| _60_ | _NCT03245151_ | _2017_ | _United States, Canada_ | _Interventional_ | _Phase I/II_ | _1: FGFR1, 2: FGFR2, 3: FGFR3, 4: FGFR4, 6: PDGFRα, 7: PDGFRβ, 8: RET, 9: VEGFR1, 10: VEGFR2, 11: VEGFR3, 12: mTORC1, 13: MTOR_ | _Both_ | _2 Years - 21 Years_ |
| _61_ | _NCT03983369_ | _2019_ | _France_ | _Interventional_ | _Phase 3 Clinical_ | _NA_ | _Both_ | _4 Years - 25 Years_ |
| _62_ | _NCT02094625_ | _2014_ | _United States_ | _Interventional_ | _Phase 1 Clinical_ | _NA_ | _Both_ | _1 Year - 21 Years_ |
| _63_ | _NCT04612504_ | _2020_ | _China_ | _Interventional_ | _Phase 1 Clinical_ | _NA_ | _Both_ | _18 Years - No limit_ |
| _64_ | _NCT05693519_ | _2023_ | _Netherlands_ | _Observation_ | _NA_ | _NA_ | _Both_ | _0 Years - 24 Years_ |
| _65_ | _NCT03245450_ | _2017_ | _Spain, United Kingdom, Germany_ | _Interventional_ | _Phase I/II_ | _1: TOP1_ | _Both_ | _6 Months - 25 Years_ |
| _66_ | _NCT02557750_ | _2015_ | _Egypt_ | _Observation_ | _NA_ | _NA_ | _Both_ | _No limit - 18 Years_ |
| _67_ | _NCT05322187_ | _2022_ | _China_ | _Interventional_ | _Phase I/II_ | _NA_ | _Both_ | _5 Years - 14 Years_ |
| _68_ | _NCT05170282_ | _2021_ | _China_ | _Observation_ | _NA_ | _NA_ | _Both_ | _0 Months - 12 Months_ |
| _69_ | _NCT03071094_ | _2017_ | _France_ | _Interventional_ | _Phase I/II_ | _NA_ | _Both_ | _18 Years - No limit_ |
| _70_ | _NCT05076071_ | _2021_ | _China_ | _Observation_ | _NA_ | _NA_ | _Both_ | _0 Years - 18 Years_ |
| _71_ | _NCT04132882_ | _2019_ | _Turkey_ | _NA_ | _NA_ | _NA_ | _Both_ | _1 Month - 18 Years_ |
| _72_ | _NCT03645655_ | _2018_ | _China_ | _Observation_ | _NA_ | _NA_ | _Both_ | _1 Month - 12 Months_ |
| _73_ | _NCT02425397_ | _2015_ | _France_ | _Observation_ | _NA_ | _NA_ | _Both_ | _4 Years - No limit_ |
| _74_ | _NCT04173676_ | _2019_ | _China_ | _Interventional_ | _Phase 1 Clinical_ | _NA_ | _Both_ | _18 Years - 75 Years_ |
| _75_ | _NCT02562755_ | _2015_ | _Portugal, Germany, New Zealand_ | _Interventional_ | _Phase 3 Clinical_ | _NA_ | _Both_ | _18 Years - No limit_ |
| _76_ | _NCT01625351_ | _2012_ | _United States_ | _Interventional_ | _Phase 1 Clinical_ | _NA_ | _Both_ | _2 Years - 21 Years_ |
| _77_ | _NCT03409341_ | _2018_ | _United States_ | _Observation_ | _NA_ | _NA_ | _Both_ | _18 Years - 70 Years_ |
| _78_ | _NCT02997189_ | _2016_ | _United States_ | _Interventional_ | _Phase 2 Clinical_ | _NA_ | _Both_ | _6 Months - 21 Years_ |
| _79_ | _NCT04400851_ | _2020_ | _China_ | _Interventional_ | _Phase 1 Clinical_ | _1: PD-1_ | _Both_ | _1 Year - 18 Years_ |
| _80_ | _NCT01956669_ | _2013_ | _Spain, Canada, Czechia_ | _Interventional_ | _Phase 2 Clinical_ | _NA_ | _Both_ | _1 Year - 18 Years_ |
| _81_ | _NCT03362814_ | _2017_ | _China_ | _Interventional_ | _Phase II/III_ | _NA_ | _Both_ | _18 Years - No limit_ |
| _82_ | _NCT01331135_ | _2011_ | _United States_ | _Interventional_ | _Phase 1 Clinical_ | _NA_ | _Both_ | _No limit - 30 Years_ |
| _83_ | _NCT04406870_ | _2020_ | _China_ | _Interventional_ | _Phase IV_ | _NA_ | _Both_ | _1 Month - 36 Months_ |
| _84_ | _NCT04093648_ | _2019_ | _—_ | _Interventional_ | _Phase 1 Clinical_ | _NA_ | _Both_ | _1 Year - No limit_ |
| _85_ | _NCT01154816_ | _2010_ | _United States, Canada_ | _Interventional_ | _Phase 2 Clinical_ | _NA_ | _Both_ | _1 Year - 21 Years_ |
| _86_ | _NCT02390843_ | _2015_ | _United States_ | _Interventional_ | _Phase 1 Clinical_ | _1: HMGCR, 2: TOP1_ | _Both_ | _1 Year - 29 Years_ |
| _87_ | _NCT03913416_ | _2019_ | _France_ | _Interventional_ | _NA_ | _NA_ | _Both_ | _1 Day - 24 Months_ |
| _88_ | _NCT00007813_ | _2004_ | _United States_ | _Interventional_ | _Phase 1 Clinical_ | _NA_ | _Both_ | _No limit - 35 Years_ |
| _89_ | _NCT02733809_ | _2016_ | _Saudi Arabia_ | _Interventional_ | _Phase IV_ | _NA_ | _Both_ | _18 Years - No limit_ |
| _90_ | _NCT04012021_ | _2019_ | _Italy_ | _Interventional_ | _NA_ | _NA_ | _Both_ | _18 Years - No limit_ |
| _91_ | _NCT01829347_ | _2013_ | _Spain, United States, United Kingdom_ | _Interventional_ | _Phase 3 Clinical_ | _NA_ | _Both_ | _18 Years - No limit_ |
| _92_ | _NCT03728543_ | _2018_ | _Russian Federation_ | _Interventional_ | _Phase II/III_ | _NA_ | _Both_ | _No limit - 18 Years_ |
| _93_ | _NCT02933333_ | _2016_ | _China_ | _Interventional_ | _Phase IV_ | _NA_ | _Both_ | _1 Year - 18 Years_ |
| _94_ | _NCT00945126_ | _2009_ | _Germany_ | _Interventional_ | _Phase 2 Clinical_ | _NA_ | _Both_ | _No limit - 30 Years_ |
| _95_ | _NCT02011126_ | _2013_ | _—_ | _Interventional_ | _Phase 2 Clinical_ | _NA_ | _Both_ | _1 Year - 30 Years_ |
| _96_ | _NCT00652132_ | _2008_ | _United Kingdom_ | _Interventional_ | _Phase 3 Clinical_ | _NA_ | _Both_ | _1 Month - 18 Years_ |
| _97_ | _NCT02164097_ | _2014_ | _United States_ | _Interventional_ | _Phase 1 Clinical_ | _NA_ | _Both_ | _1 Year - 21 Years_ |
| _98_ | _NCT00623077_ | _2008_ | _United States_ | _Interventional_ | _Phase 1 Clinical_ | _NA_ | _Both_ | _No limit - 70 Years_ |
| _99_ | _NCT00003926_ | _2003_ | _United States_ | _Interventional_ | _Phase 1 Clinical_ | _NA_ | _Both_ | _1 Year - 45 Years_ |
| _100_ | _NCT02653196_ | _2016_ | _United States_ | _Interventional_ | _Phase 0_ | _NA_ | _Both_ | _No limit - 60 Years_ |
| _101_ | _NCT01125800_ | _2010_ | _United Kingdom, Canada, Australia_ | _Interventional_ | _Phase II/III_ | _NA_ | _Both_ | _12 Months - 18 Years_ |
| _102_ | _NCT01336881_ | _2011_ | _United States_ | _Observation_ | _NA_ | _NA_ | _Both_ | _No limit - 16 Years_ |
| _103_ | _NCT01745731_ | _2012_ | _Spain_ | _Interventional_ | _Phase 2 Clinical_ | _NA_ | _Both_ | _18 Years - No limit_ |
| _104_ | _NCT00024258_ | _2003_ | _United States_ | _Interventional_ | _Phase 2 Clinical_ | _NA_ | _Both_ | _No limit - 40 Years_ |
| _105_ | _NCT01132547_ | _2010_ | _United States_ | _Interventional_ | _Phase 3 Clinical_ | _NA_ | _Both_ | _2 Years - 21 Years_ |
| _106_ | _NCT00831844_ | _2009_ | _United States, Australia, Canada_ | _Interventional_ | _Phase 2 Clinical_ | _NA_ | _Both_ | _7 Months - 30 Years_ |
| _107_ | _NCT00624962_ | _2008_ | _—_ | _Interventional_ | _NA_ | _NA_ | _Both_ | _1 Year - 21 Years_ |
| _108_ | _NCT00077389_ | _2004_ | _Ireland, United Kingdom, France_ | _Interventional_ | _Phase 2 Clinical_ | _NA_ | _Both_ | _No limit - 17 Years_ |
| _109_ | _NCT00003912_ | _2003_ | _Italy_ | _Interventional_ | _Phase 3 Clinical_ | _NA_ | _Both_ | _No limit - 16 Years_ |
| _110_ | _NCT00228683_ | _2005_ | _United States_ | _Interventional_ | _NA_ | _NA_ | _-_ | _6 Months - 21 Years_ |
| _111_ | _NCT00287976_ | _2006_ | _Ireland, United Kingdom, Netherlands_ | _Interventional_ | _Phase 2 Clinical_ | _NA_ | _Both_ | _No limit - 20 Years_ |
| _112_ | _NCT00003994_ | _2003_ | _United States_ | _Interventional_ | _Phase 3 Clinical_ | _NA_ | _Both_ | _No limit - 21 Years_ |
| _113_ | _NCT00091182_ | _2004_ | _United States_ | _Interventional_ | _Phase 2 Clinical_ | _NA_ | _Both_ | _No limit - 21 Years_ |
| _114_ | _NCT01533779_ | _2012_ | _Israel_ | _Observation_ | _NA_ | _NA_ | _Both_ | _No limit - 21 Years_ |
| _115_ | _NCT00179816_ | _2005_ | _United States_ | _Interventional_ | _Phase IV_ | _NA_ | _Both_ | _No limit - 21 Years_ |
| _116_ | _NCT03245151_ | _2022_ | _Canada, United States_ | _Interventional_ | _Phase 2 Clinical_ | _VEGFR1, mTOR_ | _Women_ | _0-17y, 2-11y, 12-17y, 18-64y_ |
| _117_ | _NBK151/1/2022_ | _2023_ | _Poland_ | _Interventional_ | _Phase 3 Clinical_ | _DNA, Topoisomerase II, Thymidylate synthetase, tubulin, Receptor tyrosine kinase_ | _Women_ | _0-17y, <37 weeks, 0-27d, 28d-23m, 2-11y, 12-17y_ |
| _118_ | _NCT02562755_ | _2014_ | _Australia, Canada, China_ | _Interventional_ | _Phase 3 Clinical_ | _Tumor cells and related cells in the tumor microenvironment, VEGFR1_ | _Women_ | _18-64y, 65+y_ |
| _119_ | _AIFA-TRS-2018-00001238_ | _2019_ | _Italy_ | _Interventional_ | _Phase IV_ | _VEGFR1_ | _Women_ | _18-64y, 65+y_ |
| _120_ | _NCT03071094_ | _2016_ | _France, Italy, United States_ | _Interventional_ | _Phase I/II_ | _PD-1_ | _Women_ | _18-64y, 65+y_ |
| _121_ | _NCT03017326_ | _2016_ | _Belgium, Czech Republic, Finland_ | _Interventional_ | _Phase 3 Clinical_ | _DNA, Topoisomerase II, Thymine nucleotide synthetase, Ribonucleotide reductase, VEGFR1, tubulin_ | _Both_ | _0-17y, <37w, 0-27d, 28d-23m, 2-11y, 12-17y, 18-64y_ |
| _122_ | _NCT03017326_ | _2016_ | _Belgium, Czech Republic, France_ | _Interventional_ | _Phase 3 Clinical_ | _DNA, Topoisomerase II, Thymine nucleotide synthetase, Ribonucleotide reductase, VEGFR1, tubulin_ | _Women_ | _0-17y, <37w, 0-27d, 28d-23m, 2-11y, 12-17y, 18-64y_ |
| _123_ | _ADVL1622_ | _2019_ | _United States_ | _Interventional_ | _Phase 2 Clinical_ | _HGFR_ | _Women_ | _0-17y, 2-11y, 12-17y, 18-64y_ |
| _124_ | _NCT03017326_ | _2016_ | _Belgium, Czech Republic, France_ | _Interventional_ | _Phase 3 Clinical_ | _DNA, Topoisomerase II, Thymine nucleotide synthetase, Ribonucleotide reductase, VEGFR1, tubulin_ | _Both_ | _0-17y, <37w, 0-27d, 28d-23m, 2-11y, 12-17y, 18-64y_ |
| _125_ | _NCT03017326_ | _2016_ | _UK, Ireland, Spain_ | _Interventional_ | _Phase 3 Clinical_ | _DNA, Topoisomerase II, Thymine nucleotide synthetase, Ribonucleotide reductase, VEGFR1, tubulin_ | _Both_ | _0-17y, <37w, 0-27d, 28d-23m, 2-11y, 12-17y_ |
| _126_ | _NCT03017326_ | _2016_ | _Belgium, Czechia, France_ | _Interventional_ | _Phase 3 Clinical_ | _DNA, Topoisomerase II, Thymine nucleotide synthetase, Ribonucleotide reductase, VEGFR1, tubulin_ | _Both_ | _0-17y, <37w, 0-27d, 28d-23m, 2-11y, 12-17y, 18-64y_ |
| _127_ | _NCT03017326_ | _2016_ | _Belgium, Czech Republic, France_ | _Interventional_ | _Phase 3 Clinical_ | _DNA, Topoisomerase II, Thymine nucleotide synthetase, Ribonucleotide reductase, VEGFR1, tubulin_ | _Both_ | _0-17y, <37w, 0-27d, 28d-23m, 2-11y, 12-17y, 18-64y_ |
| _128_ | _NCT03017326_ | _2016_ | _Belgium, Czech Republic, France_ | _Interventional_ | _Phase 3 Clinical_ | _DNA, Topoisomerase II, Thymine nucleotide synthetase, Ribonucleotide reductase, VEGFR1, tubulin_ | _Both_ | _0-17y, <37w, 0-27d, 28d-23m, 2-11y, 12-17y, 18-64y_ |
| _129_ | _NCT03017326_ | _2016_ | _Belgium, Czech Republic, France_ | _Interventional_ | _Phase 3 Clinical_ | _DNA, tubulin, Topoisomerase II, Thymidylate synthetase, VEGFR1, Ribonucleotide reductase_ | _Both_ | _0-17y, <37w, 0-27d, 28d-23m, 2-11y, 12-17y, 18-64y_ |
| _130_ | _NCT03017326_ | _2016_ | _Belgium, Czech Republic, France_ | _Interventional_ | _Phase 3 Clinical_ | _DNA, Topoisomerase II, Thymine nucleotide synthetase, Ribonucleotide reductase, VEGFR1, tubulin_ | _Women_ | _0-17y, <37w, 0-27d, 28d-23m, 2-11y, 12-17y, 18-64y_ |
| _131_ | _NCT02562755_ | _2014_ | _Australia, Canada, China_ | _Interventional_ | _Phase 3 Clinical_ | _VEGFR1_ | _Women_ | _18-64y, 65+y_ |
| _132_ | _VEG116731/CPZP034X2203_ | _2013_ | _Canada, Czech Republic, France_ | _Interventional_ | _Phase 2 Clinical_ | _VEGFR1_ | _Women_ | _0-17y, 2-11y_ |
| _133_ | _VEG116731/CPZP034X2203_ | _2013_ | _Canada, Czech Republic, France_ | _Interventional_ | _Phase 2 Clinical_ | _VEGFR1_ | _Women_ | _0-17y, 28d-23m, 2-11y, 12-17y, 18-64y_ |
| _134_ | _VEG116731/CPZP034X2203_ | _2013_ | _Canada, Czech Republic, France_ | _Interventional_ | _Phase 2 Clinical_ | _VEGFR1_ | _Women_ | _0-17y, 28d-23m, 2-11y, 12-17y, 18-64y_ |
| _135_ | _NCT03017326_ | _2016_ | _Belgium, Czech Republic, France_ | _Interventional_ | _Phase 3 Clinical_ | _DNA, Topoisomerase II, Thymine nucleotide synthetase, Ribonucleotide reductase, VEGFR1, tubulin_ | _Both_ | _0-17y, <37w, 0-27d, 28d-23m, 2-11y, 12-17y, 18-64y_ |
| _136_ | _BAY73-4506/15906_ | _2013_ | _Italy, UK, France_ | _Interventional_ | _Phase 1 Clinical_ | _VEGFR-1_ | _Women_ | _0-17y, 28d-23m, 2-11y, 12-17y_ |
| _137_ | _VEG116731/CPZP034X2203_ | _2013_ | _Canada, Czech Republic, France_ | _Interventional_ | _Phase 2 Clinical_ | _VEGFR1_ | _Women_ | _0-17y, 2-11y_ |
| _138_ | _VEG116731/PZP034X2203_ | _2013_ | _Canada, Czech Republic, France_ | _Interventional_ | _Phase 2 Clinical_ | _VEGFR1_ | _Women_ | _0-17y, 2-11y_ |
| _139_ | _NCT03017326_ | _2016_ | _Belgium, Czech Republic, France_ | _Interventional_ | _Phase 3 Clinical_ | _DNA, Topoisomerase II, Thymine nucleotide synthetase, Ribonucleotide reductase, VEGFR1, tubulin_ | _Both_ | _0-17y, <37w, 0-27d, 28d-23m, 2-11y, 12-17y, 18-64y_ |
| _140_ | _NCT03017326_ | _2016_ | _Belgium, France, Germany_ | _Interventional_ | _Phase 3 Clinical_ | _DNA, Topoisomerase II, Thymine nucleotide synthetase, Ribonucleotide reductase, VEGFR1, tubulin_ | _Both_ | _0-17y, <37w, 0-27d, 28d-23m, 2-11y, 12-17y, 18-64y_ |
| _141_ | _NCT02562755_ | _2014_ | _Australia, Canada, China_ | _Interventional_ | _Phase 3 Clinical_ | _VEGFR1_ | _Women_ | _18-64y, 65+y_ |
| _142_ | _GETHI021_ | _2016_ | _Spain_ | _Interventional_ | _Phase 2 Clinical_ | _PD-1_ | _Both_ | _18-64y, 65+y_ |
| _143_ | _NCT03017326_ | _2016_ | _Belgium, Czech Republic, France_ | _Interventional_ | _Phase 3 Clinical_ | _DNA, Topoisomerase II, Thymine nucleotide synthetase, Ribonucleotide reductase, VEGFR1, tubulin_ | _Both_ | _0-17y, <37w, 0-27d, 28d-23m, 2-11y, 12-17y, 18-64y_ |
| _144_ | _NCT02612428_ | _2015_ | _Austria, Germany, Ireland_ | _Interventional_ | _Phase 3 Clinical_ | _NA_ | _Women_ | _18-64y_ |
| _145_ | _NCT02562755_ | _2014_ | _Australia, Canada, China_ | _Interventional_ | _Phase 3 Clinical_ | _VEGFR1_ | _Women_ | _18-64y, 65+y_ |
| _146_ | _NCT02562755_ | _2014_ | _Australia, Canada, China_ | _Interventional_ | _Phase 3 Clinical_ | _VEGFR1_ | _Women_ | _18-64y, 65+y_ |
| _147_ | _VTL-308_ | _2015_ | _Germany, Ireland, Spain_ | _Interventional_ | _Phase 3 Clinical_ | _NA_ | _Women_ | _18-64y_ |
| _148_ | _VTL-308_ | _2015_ | _Germany, Ireland, Spain_ | _Interventional_ | _Phase 3 Clinical_ | _NA_ | _Women_ | _18-64y_ |
| _149_ | _VTL-308_ | _2015_ | _Germany, Ireland, Spain_ | _Interventional_ | _Phase 3 Clinical_ | _NA_ | _Women_ | _18-64y_ |
| _150_ | _NCT01829347_ | _2013_ | _Austria, Germany, Ireland_ | _NA_ | _Phase 3 Clinical_ | _NA_ | _Women_ | _18-64y, 65+y_ |
| _151_ | _BAY73-4506/15906_ | _2013_ | _Italy, UK, France_ | _Interventional_ | _Phase 1 Clinical_ | _VEGFR-1_ | _Women_ | _0-17y, 28d-23m, 2-11y, 12-17y_ |
| _152_ | _ADVL1322-VEG116731_ | _2013_ | _Canada, United States_ | _Interventional_ | _Phase 2 Clinical_ | _VEGFR-1_ | _Both_ | _0-17y, 28d-23m, 2-11y, 12-17y, 18-64y_ |
| _153_ | _VTI-210_ | _2013_ | _Australia, Germany, Spain_ | _Interventional_ | _Phase 3 Clinical_ | _NA_ | _Women_ | _18-64y_ |
| _154_ | _VTI-208_ | _2012_ | _Australia, Germany, New Zealand_ | _Interventional_ | _Phase 3 Clinical_ | _NA_ | _Women_ | _18-64y, 65+y_ |
| _155_ | _NCT01829347_ | _2013_ | _Australia, Germany, Spain_ | _Interventional_ | _Phase 3 Clinical_ | _NA_ | _Women_ | _18-64y_ |
| _156_ | _NCT01829347_ | _2013_ | _Germany, Spain, United States_ | _Interventional_ | _Phase 3 Clinical_ | _NA_ | _Women_ | _18-64y, 65+y_ |
| _157_ | _VTI-208_ | _2012_ | _Australia, Germany, Spain_ | _Interventional_ | _Phase 3 Clinical_ | _NA_ | _Women_ | _18-64y, 65+y_ |
| _158_ | _LT200703_ | _2007_ | _UK, Belgium, France_ | _Interventional_ | _Phase 3 Clinical_ | _DNA_ | _Women_ | _0-17y, 28d-23m, 2-11y, 12-17y, 18-64y_ |
| _159_ | _NCT01471028_ | _2012_ | _Australia, Spain, United States_ | _Interventional_ | _Phase 3 Clinical_ | _NA_ | _Women_ | _18-64y, 65+y_ |
| _160_ | _JX594-IV-HEP021_ | _2012_ | _Hong Kong, Korea, United States_ | _Interventional_ | _Phase 2 Clinical_ | _NA_ | _Women_ | _18-64y, 65+y_ |
| _161_ | _NCT01387555_ | _2011_ | _Australia, Canada, Hong Kong_ | _Interventional_ | _Phase 2 Clinical_ | _NA_ | _Women_ | _18-64y, 65+y_ |
| _162_ | _SIOPEL6_ | _2007_ | _Australia_ | _Interventional_ | _Phase 3 Clinical_ | _Cyanide ion (CN⁻)_ | _Both_ | _0-17y, 28d-23m, 2-11y, 12-17y_ |
| _163_ | _CLDE225X2104_ | _2010_ | _France, UK, Italy_ | _Interventional_ | _Phase 1 Clinical_ | _NA_ | _Women_ | _0-17y, 28d-23m, 2-11y, 12-17y_ |
| _164_ | _NCT01125800_ | _2010_ | _Australia, Canada, United States_ | _Interventional_ | _Phase I/II_ | _Smoothened protein_ | _Women_ | _0-17y, 28d-23m, 2-11y, 12-17y_ |
| _165_ | _CLDE225X2104_ | _2010_ | _France, UK, Italy_ | _Interventional_ | _Phase 1 Clinical_ | _Smoothened protein_ | _Women_ | _0-17y, 28d-23m, 2-11y, 12-17y_ |
| _166_ | _CMMo/RH/2009_ | _2009_ | _Spain_ | _Interventional_ | _Phase 2 Clinical_ | _NA_ | _Both_ | _18-64y, 65+y_ |
| _167_ | _RG_09-205_ | _2007_ | _UK, Belgium, France_ | _Interventional_ | _Phase 3 Clinical_ | _DNA_ | _Women_ | _0-17y, 28d-23m, 2-11y, 12-17y, 18-64y_ |
| _168_ | _VTI-206_ | _2009_ | _United States_ | _Interventional_ | _Phase 2 Clinical_ | _NA_ | _Women_ | _18-64y, 65+y_ |
| _169_ | _P/2008/69_ | _2007_ | _UK, Belgium, France_ | _Interventional_ | _Phase 3 Clinical_ | _Cyanide ion (CN⁻)_ | _Women_ | _0-17y, 28d-23m, 2-11y, 12-17y, 18-64y_ |
| _170_ | _LT 2007 03_ | _2007_ | _UK, Belgium, France_ | _Interventional_ | _Phase 3 Clinical_ | _Cyanide ion (CN⁻)_ | _Women_ | _0-17y, 28d-23m, 2-11y, 12-17y, 18-64y_ |
| _171_ | _RG_09-205_ | _2007_ | _UK, Belgium, France_ | _Interventional_ | _Phase 3 Clinical_ | _Cyanide ion (CN⁻)_ | _Women_ | _0-17y, 28d-23m, 2-11y, 12-17y, 18-64y_ |
| _172_ | _SIOPEL 4_ | _2006_ | _Ireland, Belgium_ | _Interventional_ | _Phase 2 Clinical_ | _DNA, Topoisomerase II_ | _Women_ | _0-17y, 0-27d, 28d-23m, 2-11y, 12-17y_ |
| _173_ | _LT 2004 09_ | _2006_ | _Ireland, Belgium_ | _Interventional_ | _Phase 2 Clinical_ | _DNA, Topoisomerase II_ | _Women_ | _0-17y, 0-27d, 28d-23m, 2-11y, 12-17y_ |
| _174_ | _200605_ | _2005_ | _Austria_ | _Interventional_ | _Phase 2 Clinical_ | _Topoisomerase I_ | _Women_ | _0-17y, 28d-23m, 2-11y, 12-17y_ |
| _175_ | _05LG12_ | _2005_ | _UK_ | _Interventional_ | _Phase IV_ | _IMPDH_ | _Women_ | _0-17y, 2-11y, 12-17y_ |
| _176_ | _UMIN000056899_ | _2025_ | _Japan_ | _Observation_ | _NA_ | _NA_ | _Both_ | _No limit - 18 years_ |
| _177_ | _jRCTs061180004_ | _2018_ | _Singapore, Japan_ | _Interventional_ | _Phase 2 Clinical_ | _NA_ | _Both_ | _0 - 30 years_ |
| _178_ | _jRCT2061220018_ | _2022_ | _Japan_ | _Interventional_ | _Phase 2 Clinical_ | _NA_ | _Both_ | _1 month - 30 years_ |
| _179_ | _jRCT1060240085_ | _2024_ | _Japan_ | _Observation_ | _NA_ | _NA_ | _Both_ | _No limit - 40 years_ |
| _180_ | _UMIN000055367_ | _2024_ | _Japan_ | _Observation_ | _NA_ | _NA_ | _Both_ | _1 day - 90 years_ |
| _181_ | _UMIN000054107_ | _2024_ | _Japan_ | _Interventional_ | _NA_ | _NA_ | _Children_ | _4 years - 7 years_ |
| _182_ | _jRCT2073220054_ | _2022_ | _Japan_ | _Interventional_ | _Phase 1 Clinical_ | _NA_ | _Both_ | _1 - 24 years_ |
| _183_ | _UMIN000019840_ | _2015_ | _Japan, North America_ | _Interventional_ | _Phase 3 Clinical_ | _NA_ | _Both_ | _No limit - 21 years_ |
| _184_ | _UMIN000022324_ | _2016_ | _Japan_ | _Interventional_ | _NA_ | _NA_ | _Both_ | _No limit - 18 years_ |
| _185_ | _UMIN000021931_ | _2016_ | _Japan_ | _Interventional_ | _NA_ | _NA_ | _Both_ | _No limit - 18 years_ |
| _186_ | _UMIN000017453_ | _2015_ | _Japan_ | _Interventional_ | _Phase 1 Clinical_ | _NA_ | _Both_ | _1 day - 18 years_ |
| _187_ | _UMIN000009049_ | _2012_ | _Japan_ | _Interventional_ | _Phase 2 Clinical_ | _NA_ | _Both_ | _1 day - 18 years_ |
| _188_ | _UMIN000016305_ | _2015_ | _Japan_ | _Interventional_ | _Phase I/II_ | _NA_ | _Both_ | _1 day - 18 years_ |
| _189_ | _UMIN000009050_ | _2012_ | _Japan_ | _Interventional_ | _Phase 2 Clinical_ | _NA_ | _Both_ | _1 month - 196 months_ |
| _190_ | _jRCTs061180086_ | _2019_ | _Japan_ | _Interventional_ | _Phase 2 Clinical_ | _NA_ | _Both_ | _1 month - 18 years_ |
| _191_ | _UMIN000001116_ | _2008_ | _Japan_ | _Observation_ | _Phase 2 Clinical_ | _NA_ | _Both_ | _1 day - 15 years_ |
| _192_ | _UMIN000009048_ | _2012_ | _Japan, Asia (except Japan), Australia_ | _Interventional_ | _Phase 3 Clinical_ | _NA_ | _Both_ | _1 month - 227 months_ |
| _193_ | _UMIN000020144_ | _2015_ | _Japan_ | _Interventional_ | _Phase I/II_ | _NA_ | _Both_ | _1 year - 40 years_ |
| _194_ | _UMIN000026474_ | _2017_ | _Japan_ | _Interventional_ | _NA_ | _NA_ | _Both_ | _NA_ |
| _195_ | _UMIN000003002_ | _2010_ | _Japan_ | _Interventional_ | _Phase 2 Clinical_ | _NA_ | _Both_ | _3 years - 30 years_ |
| _196_ | _UMIN000001037_ | _2008_ | _Japan_ | _Interventional_ | _Phase I/II_ | _NA_ | _Both_ | _1 year - 30 years_ |
| _197_ | _ISRCTN17869351_ | _2001_ | _Austria, Belgium, Czech Republic_ | _Interventional_ | _NA_ | _NA_ | _Both_ | _0 Years - 30 Years_ |
| _198_ | _ISRCTN87675276_ | _2016_ | _England, United Kingdom_ | _Interventional_ | _NA_ | _DNA, Topoisomerase II_ | _Both_ | _0 Years - 16 Years_ |
| _199_ | _ISRCTN16342840_ | _2011_ | _Brazil_ | _Interventional_ | _NA_ | _NA_ | _Both_ | _18 Years - No Limit_ |
| _200_ | _ISRCTN64076941_ | _2024_ | _Spain_ | _Interventional_ | _NA_ | _NA_ | _Both_ | _18 Years - No Limit_ |
| _201_ | _CTRI/2024/03/064371_ | _2024_ | _India_ | _Interventional_ | _Phase 3 Clinical_ | _Dopamine receptor_ | _Children_ | _2 - 18 Years_ |
| _202_ | _CTRI/2024/06/069360_ | _2024_ | _India_ | _Observation_ | _NA_ | _NA_ | _Children_ | _1 Day - 12 Years_ |
| _203_ | _CTRI/2023/03/051221_ | _2023_ | _India_ | _Observation_ | _NA_ | _NA_ | _Children_ | _3 - 17 Years_ |
| _204_ | _CTRI/2022/07/044087_ | _2022_ | _India_ | _Interventional_ | _-_ | _NA_ | _Children_ | _1 - 10 Years_ |
| _205_ | _CTRI/2021/03/032430_ | _2021_ | _India_ | _Interventional_ | _NA_ | _NA_ | _Both_ | _18 - 80 Years_ |
| _206_ | _CTRI/2017/02/007925_ | _2017_ | _India_ | _Interventional_ | _Phase 3 Clinical_ | _NK-1_ | _Children_ | _1 - 12 Years_ |
| _207_ | _CTRI/2021/05/033611_ | _2021_ | _India_ | _Observation_ | _NA_ | _NA_ | _Children_ | _0 - 18 Years_ |
| _208_ | _KCT0000519_ | _2012_ | _Korea_ | _Interventional_ | _Phase 2 Clinical_ | _NA_ | _Both_ | _18 Years - No Limit_ |
| _209_ | _DRKS00007623_ | _2014_ | _Austria, Belgium, Czechia_ | _Observation_ | _NA_ | _NA_ | _-_ | _No limit - 40 Years_ |
| _210_ | _DRKS00027082_ | _2021_ | _Germany_ | _Observation_ | _NA_ | _NA_ | _-_ | _18 Years - No limit_ |
| _211_ | _DRKS00024485_ | _2022_ | _Germany_ | _Observation_ | _NA_ | _NA_ | _-_ | _18 Years - No limit_ |
| _212_ | _DRKS00023554_ | _2021_ | _Germany_ | _Observation_ | _NA_ | _NA_ | _-_ | _18 Years - 100 Years_ |
| _213_ | _DRKS00010427_ | _2016_ | _Germany_ | _Interventional_ | _NA_ | _NA_ | _-_ | _18 Years - No limit_ |
| _214_ | _TCTR20220418005_ | _2022_ | _India_ | _Observation_ | _NA_ | _NA_ | _-_ | _2 Months - 60 Months_ |
| _215_ | _TCTR20210221001_ | _2021_ | _Thailand_ | _Observation_ | _NA_ | _NA_ | _-_ | _1 Year - 15 Years_ |
| _216_ | _219451_ | _2024_ | _Argentina, Czechia, Spain_ | _Interventional_ | _-_ | _TIM-3, PD-1_ | _-_ | _18-64y, 0-17y, 0-27d, 6-11y, 12-17y, 28d-23m, 2-5y_ |
| _217_ | _NCT03017326_ | _2024_ | _Norway, Switzerland, United Kingdom_ | _Interventional_ | _Phase 3 Clinical_ | _RAF kinase, DNA, Thymidylate synthase, Topoisomerase II, Topoisomerase I, tubulin_ | _-_ | _18-64y, 0-17y, 28d-23m, 0-27d, 12-17y, 6-11y, Preterm, 2-5y_ |
| _218_ | _NBK151/1/2022_ | _2024_ | _Poland_ | _Interventional_ | _Phase 3 Clinical_ | _RAF kinase, DNA, Thymidylate synthase, Topoisomerase II, Topoisomerase I, tubulin_ | _-_ | _18-64y, 0-17y_ |
| _219_ | _NL-OMON50638_ | _2019_ | _Netherlands_ | _Interventional_ | _Phase 3 Clinical_ | _DNA, tubulin, Topoisomerase II, Thymidylate synthase_ | _-_ | _16-17y, 2-11y, 65+y, 12-15y, 18-64y_ |

**Supplementary Dataset S2**: The specific distribution of publication years

| **Year** | **value** |
| --- | --- |
| 2005 | 90 |
| 2006 | 82 |
| 2007 | 89 |
| 2008 | 85 |
| 2009 | 92 |
| 2010 | 87 |
| 2011 | 91 |
| 2012 | 127 |
| 2013 | 127 |
| 2014 | 115 |
| 2015 | 112 |
| 2016 | 116 |
| 2017 | 135 |
| 2018 | 150 |
| 2019 | 182 |
| 2020 | 163 |
| 2021 | 212 |
| 2022 | 183 |
| 2023 | 196 |
| 2024 | 201 |
